# Susceptibility and Permissivity of Zebrafish (*Danio rerio*) Larvae to Cypriniviruses

**DOI:** 10.3390/v15030768

**Published:** 2023-03-17

**Authors:** Cindy Streiff, Bo He, Léa Morvan, Haiyan Zhang, Natacha Delrez, Mickael Fourrier, Isabelle Manfroid, Nicolás M. Suárez, Stéphane Betoulle, Andrew J. Davison, Owen Donohoe, Alain Vanderplasschen

**Affiliations:** 1 Immunology–Vaccinology, Department of Infectious and Parasitic Diseases, Fundamental and Applied Research for Animals & Health (FARAH), Faculty of Veterinary Medicine, University of Liège, B-4000 Liège, Belgium; 2 Zebrafish Development and Disease Models Laboratory, GIGA-Molecular Biology of Diseases, University of Liège, B-4000 Liège, Belgium; 3 MRC-University of Glasgow Centre for Virus Research, Glasgow G61 1QH, UK; 4 UMR-I 02 Stress Environnementaux et BIOsurveillance des Milieux Aquatiques (SEBIO), UFR Sciences Exactes et Naturelles, Université de Reims Champagne-Ardenne, CEDEX 2, 51687 Reims, France; 5 Bioscience Research Institute, Technological University of the Shannon, N37 HD68 Athlone, Co. Westmeath, Ireland

**Keywords:** anguillid herpesvirus 1, cyprinid herpesvirus 2, cyprinid herpesvirus 3, alloherpesvirus, cyprinivirus, zebrafish, PKR, PKZ, CRISPR/Cas9, innate immunity

## Abstract

The zebrafish (*Danio rerio*) represents an increasingly important model organism in virology. We evaluated its utility in the study of economically important viruses from the genus *Cyprinivirus* (anguillid herpesvirus 1, cyprinid herpesvirus 2 and cyprinid herpesvirus 3 (CyHV-3)). This revealed that zebrafish larvae were not susceptible to these viruses after immersion in contaminated water, but that infections could be established using artificial infection models in vitro (zebrafish cell lines) and in vivo (microinjection of larvae). However, infections were transient, with rapid viral clearance associated with apoptosis-like death of infected cells. Transcriptomic analysis of CyHV-3-infected larvae revealed upregulation of interferon-stimulated genes, in particular those encoding nucleic acid sensors, mediators of programmed cell death and related genes. It was notable that uncharacterized non-coding RNA genes and retrotransposons were also among those most upregulated. CRISPR/Cas9 knockout of the zebrafish gene encoding protein kinase R (PKR) and a related gene encoding a protein kinase containing Z-DNA binding domains (PKZ) had no impact on CyHV-3 clearance in larvae. Our study strongly supports the importance of innate immunity-virus interactions in the adaptation of cypriniviruses to their natural hosts. It also highlights the potential of the CyHV-3-zebrafish model, versus the CyHV-3-carp model, for study of these interactions.

## 1. Introduction

The zebrafish (*Danio rerio*) is a member of the family *Cyprinidae*. It is an extremely useful experimental subject due to its high fecundity and short generation time and is currently one of the most widely used laboratory animal model organisms. Also, its transparent larval stage is highly suited to in vivo imaging, making it particularly well suited to studying host-pathogen interaction, including during viral infection [1]. Furthermore, the availability of a well-annotated zebrafish reference genome [2] and large range of recombinant and mutant zebrafish lines [3] greatly facilitates investigations into gene function in various biological contexts. The zebrafish is known to possess a well-developed immune system, composed of both innate and adaptive immune responses [4,5]. Despite some notable differences and although sites of maturation differ [6], many mammalian immune system cell types have zebrafish counterparts [7,8]. Also, zebrafish orthologs of many (but not all) mammalian pathogen recognition receptors (PRRs), cytokines, adaptor proteins for signal transduction and other important components have been identified [6,9,10], indicating that zebrafish represent a relatively useful model for studying the mechanisms that vertebrates use to detect and respond to pathogen-associated molecular patterns (PAMPs).

Although juvenile and adult zebrafish utilize both the innate and the adaptive branches of the immune system, the embryonic and larval stages rely solely on innate immunity, which is detectable and active on the first day of zebrafish embryogenesis, whereas the adaptive system is fully matured by 4–6 weeks post-fertilization [11,12]. During these early life stages, cellular immunity is mediated by myeloid cells only, with macrophages and neutrophils acting as the main effector cells [13,14]. As in mammals, the zebrafish antiviral response is orchestrated by type I pathogen induced interferons (IFNs). These are named IFNϕ1, IFNϕ2, IFNϕ3, and IFNϕ4 [15] (referred to hereafter by the respective gene symbols *ifnphi1*, *ifnphi2*, *ifnphi3*, and *ifnphi4*) and are structurally similar to mammalian type I (α and β) and type III (λ) IFNs. As in all vertebrates, type I IFNs in zebrafish induce the expression of antiviral genes broadly referred to as interferon stimulated genes (ISGs). However, the IFN response in zebrafish larvae is mediated solely by *ifnphi1* and *ifnphi3*, with *ifnphi2* being expressed only in adults and with *ifnphi4* having little activity [16,17]. The zebrafish type II IFN family consists of two members, IFNγ1 and IFNγ2 which are also responsible for the induction of ISGs induced by type I IFNs [18].

Taken together, this indicates that the zebrafish represents a relevant and useful model for studying viral pathogenicity, vertebrate host immune response, and viral host-interactions. Strikingly, very few viruses are known to infect zebrafish naturally [19,20,21,22]. Moreover, despite the lower host temperature, several mammalian viruses can infect zebrafish under experimental conditions, with these hosts exhibiting varying degrees of susceptibility and permissivity to infection. This property has also been exploited to study human viruses such as influenza A virus, Chikungunya virus (CHIKV), herpes simplex virus type 1 (HSV-1) and human norovirus [23,24,25,26]. Moreover, infection of zebrafish has been explored in studying severe acute respiratory syndrome coronavirus 2 (SARS-CoV-2) [27,28].

Zebrafish can also be infected with several important fish viruses [29,30,31,32,33,34,35]. One of these, the spring viraemia of carp virus (SVCV), which is a rhabdovirus responsible for a highly contagious disease of the common carp (*Cyprinus carpio carpio*), has become one of the viruses most frequently used in infection models for studying the antiviral immune response in zebrafish larvae and adults [16,17,35,36,37]. Recent work by Rakus et al. [38] demonstrated that cyprinid herpesvirus 3 (CyHV-3) induces an abortive infection after intraperitoneal inoculation of adult zebrafish. CyHV-3 causes mass mortality in common carp and koi carp (*Cyprinus carpio koi*), resulting in massive economic losses [39]. CyHV-3 is a member of the genus *Cyprinivirus* in the family *Alloherpesviridae*, which consists of herpesviruses that infect fish and amphibians.

In addition to CyHV-3, the genus *Cyprinivirus* contains two other economically important viruses: anguillid herpesvirus 1 (AngHV-1) and cyprinid herpesvirus 2 (CyHV-2) [40]. AngHV-1 infects the European eel (*Anguilla anguilla*), Japanese eel (*Anguilla japonica*), and American eel (*Anguilla rostrata*) [41]; CyHV-2 also infects goldfish (*Carassius auratus*) and the closely related Prussian carp (*Carassius gibelio*) and crucian carp (*Carassius carassius*) [42]. Like zebrafish, the natural hosts of CyHV-2 and CyHV-3 are also members of the family *Cyprinidae*. Cypriniviruses cause diseases only in their natural host species, which suggests the existence of restrictions related to host cell susceptibility (i.e., the ability to support virus entry) and host cell permissivity (i.e., the ability to support viral replication and the transmission of viable viral progeny to new cells, although the former may occur without the latter). Notably, experiments relying on infection of cell lines have demonstrated the ability of cypriniviruses to infect, even if inefficiently, cells originating from non-natural host species. Indeed, both CyHV-2 and CyHV-3 are capable of infecting cell lines derived from species within the family *Cyprinidae* that are not their natural hosts [39,42], with CyHV-3 already known to infect zebrafish cell lines [38]. Similarly, despite not naturally infecting species outside of the family *Anguillidae*, it has been demonstrated that AngHV-1 can infect at least one cell line derived from a member of the family *Cyprinidae* [43]. These data suggest that the ability of cypriniviruses to induce diseases only in their natural host species may be related to complex host-virus interactions downstream of host cell susceptibility.

In the present study, we conducted an in-depth evaluation and comparison of AngHV-1, CyHV-2, and CyHV-3 in terms of their ability to infect zebrafish models both in vitro and in vivo. These experiments involved the exploitation of recombinant viruses expressing reporters, timelapse epifluorescence microscopy in vitro, live imaging and transcriptomics in vivo; and finally, the generation of CRISPR/Cas9 mutant hosts to investigate the potential modulation of zebrafish permissivity to infection. Our study strongly supports the importance of the innate immune response alone in clearing viral infection and emphasizes the high degree of adaptation that cypriniviruses have undergone to facilitate successful circulation within their respective natural hosts. It also highlights the potential value of the CyHV-3-zebrafish model versus CyHV-3-carp models to study the fundamental features of virus-host interactions.

## 2. Materials and Methods

### 2.1. Cells and Viruses

The zebrafish embryonic fibroblast cells line (ZF4) [44] was kindly provided by Dr K. Rakus (Department of Evolutionary Immunology, Jagiellonian University, Poland) and cultured in advanced Dulbecco’s modified Eagle’s Medium/Ham’s F-12 (Gibco, New York, NY, USA), supplemented with 10% foetal calf serum (FCS), 2% penicillin–streptomycin (Sigma-Aldrich, St. Louis, MO, USA) and 1% L-glutamine (Lonza, Basel, Switzerland). Cells were cultured at 25 °C in a humid atmosphere containing 5% CO_2_. Eel kidney (EK-1) [45], Ryukin goldfish fin (RyuF-2) [46], and common carp brain (CCB) [47] cell lines were used to produce stocks of AngHV-1, CyHV-2, and CyHV-3, respectively. These cells were cultured as described previously [46,48,49].

Three previously described recombinant viral strains were utilized. The CyHV-3 FL BAC revertant ORF136 Luc strain (referred to as CyHV-3 Luc; GenBank accession KP343683.1) was derived from the CyHV-3 FL BAC plasmid and encodes a firefly (*Photinus pyralis*) luciferase (Luc2) reporter cassette driven by a human cytomegalovirus (CMV) promoter inserted between ORF136 and ORF137 [50]. The AngHV-1 Luc-copGFP and the CyHV-2 Luc-copGFP recombinant strains both encode the same reporter genes consisting of the Luc2 cassette and a copepod (*Pontellina plumata*) GFP (copGFP) cassette linked by a T2A sequence, driven by a eukaryotic translation elongation factor 1 alpha (EF-1α) promoter. To generate these recombinants, the dual Luc2/copGFP cassette was inserted in the region between ORF32 and ORF33 in the AngHV-1 UK parental strain genome (GenBank accession MW580855.1) [40] (Delrez et al., unpublished data) and in the intergenic region between ORF64 and ORF66 in CyHV-2 YC-01 parental strain genome (GenBank accession no. MN593216.1) (He et al., unpublished data) using homologous recombination in eucaryotic cells, as described previously [50].

In addition to the three recombinant strains described above, a fourth strain, expressing enhanced (EGFP), referred to as the CyHV-3 EGFP strain, was derived from the CyHV-3 Luc strain and constructed specifically for this study. Details relating to the generation and verification of this strain are provided in Methods S1.

### 2.2. In Vitro Experiments

#### 2.2.1. Virus Infections

ZF4 cells cultured in 24-well plates were mock-infected or infected at 24 hours (h) after seeding. Virus was diluted in 0.5 mL serum-free cell culture medium to provide a multiplicity of infection (MOI) of 3 plaque forming units (PFU)/cell. After incubating for 2 h, 1 mL fresh cell culture medium was added without removing the inoculum, and the cells were incubated at 25 °C with 5% CO_2._

#### 2.2.2. Timelapse Imaging of Infected Cells

At 24 h post infection (hpi), virus-infected ZF4 cells in 24-well plates were placed in an IncuCyte Zoom HD/2CLR microscopy system (Sartorius), which was maintained at 25 °C with 5% CO_2_. Each well was imaged at 9 different fields of view every 2 h from 1–11 days post infection (dpi). Images were collected in phase contrast and in the green (GFP) channels. Each infection was done in triplicate wells.

#### 2.2.3. Image Analysis

Data from timelapse imaging of infected cells was analysed using the Fiji plugin TrackMate (v7.7.2) [51] to track fluorescent reporter expression from individual ZF4 cells infected with CyHV-2 or CyHV-3, and by extension, to identify cell infection and cell death events with respect to time. Image sequences containing 123 frames/field of view/well were generated using a series of images acquired from 1 to 11 dpi. Analysis was performed using the default settings with LoG Detector and Simple LAP Tracker. Additional parameters were adjusted empirically in order adequately to detect and monitor fluorescence from infected cells within frames (estimated object diameter: 28.6 pixels; quality threshold: 1). Data were exported in .csv format and imported into GraphPad Prism (v8.0.1) for further analysis and visualization.

### 2.3. Experiments Using Zebrafish

#### 2.3.1. Zebrafish Larvae Maintenance

Wild-type (WT, +/+) AB strain adult zebrafish (*Danio rerio*) were obtained by natural spawning and maintained at 27 °C, on a 14/10 h light/dark cycle. They were housed in the GIGA Zebrafish facility in Liège (Belgium) according to animal research guidelines and with the approval of the local ethical commission for animal care and use. Larvae were obtained by pairwise mating of adults in mating cages and maintained in petri dishes with standard embryo medium (E3) and incubated at 25 °C prior to use in experiments.

#### 2.3.2. Inoculation of Larvae by Immersion

Zebrafish larvae (3 days post-fertilization (dpf)) were placed in 24-well plates containing 1 mL E3 medium and either mock-infected or infected by immersion. For infection, virus suspensions were added to each well and mixed gently (final concentration: 4000 PFU/mL), and plates were incubated at 25 °C.

#### 2.3.3. Inoculation of Larvae by Microinjection

Borosilicate glass capillaries were loaded with 10 µL of medium containing virus suspensions (1.2 × 10^6^ PFU/mL) and then connected to a FemtoJet microinjector (Eppendorf, Framingham, MA, USA) as described elsewhere [52]. After breaking the capillary tip, the pressure was adjusted to obtain droplets with a diameter of ~0.13 mm. Larvae (3 dpf) were anesthetized in a bath containing tricaine (0.2 mg/mL). The fish were positioned on a petri dish, and the surface of the dish was dried entirely in order to avoid drifting of the larvae during viral injections. In order to visualize the hearts of the larvae, the petri dish was placed under a binocular magnifier (LEICA MZ6) at 4x magnification and illuminated by an external light source (LEICA CLS 50X). The capillary was then manually inserted into the pericardial cavity and three pulses were performed to inject approximately 3 nL of virus suspension (infected fish) or 3 nL of PBS (mock-infected fish). After microinjection, the larvae were transferred into individual wells in a 24-well plate containing 1 mL E3 medium and incubated at 25 °C.

#### 2.3.4. Epifluorescence Microscopy

The progression of infection with recombinant viruses expressing fluorescent reporters was monitored using epifluorescence microscopy. This facilitated longitudinal observation of the same larvae at multiple timepoints. Prior to observation, larvae were anesthetized in a bath of E3 medium containing tricaine (0.2 mg/mL) and methylcellulose (2% *w*/*v*) in order to avoid drifting of larvae. Imaging of larvae was performed using a Leica DM2000 epifluorescence microscope at 5× and 10× magnification. After imaging, larvae were immediately transferred back to their individual wells and returned to the incubator. After the final observation timepoint, larvae were euthanized using an overdose of tricaine in E3 media (400 mg/L).

#### 2.3.5. In Vivo Bioluminescent Imaging

An in vivo imaging (IVIS) system (IVIS Spectrum, PerkinElmer) was used to detect bioluminescence in larvae infected with Luc2-expressing recombinant viruses, thus facilitating the monitoring and quantification of viral levels in vivo. At the time of imaging, larvae were anesthetized (as described for epifluorescence microscopy analysis), injected with ~3 nL of D-luciferin (15 mg/mL), and imaged 5 minutes (min) after injection. Images were acquired using the following settings: field of view A, small binning, automatic exposure time with a maximum of 1 min and a subject height of 0.30 cm. Unlike epifluorescence analysis, longitudinal monitoring of individual larvae was not possible due to the harmful effects of repeated D-luciferin injections in the same larvae. Relative bioluminescence intensities were analysed using Living Image software (v4.7.3). Regions of interest (ROIs) were drawn by manually outlining the larval body, and bioluminescence within the ROI was recorded in terms of mean radiance (photons/s/cm^2^/sr).

#### 2.3.6. In Vivo Timelapse Imaging

For time-lapse imaging, live larvae infected with CyHV-3 EGFP were imaged using a Zeiss Z1 light sheet microscope according to the protocol described elsewhere [53]. Briefly, larvae were embedded inside FEP tubes containing 0.1% low melting point agarose and tricaine (55 µg/mL) and maintained at 27 °C. Z-stacks encompassing the entire head and heart regions were acquired every 10 min from 2 to 3 dpi and were used to generate a maximum-intensity projection video with ImageJ.

#### 2.3.7. Ethics Statement

The experiments performed in the present study did not require a bioethical permit as they involved the use of larvae before implementation of feeding. However, all experiments were designed and conducted in accord with the 3R rules and other bioethics standards.

### 2.4. RNA-Seq Analysis

#### 2.4.1. Zebrafish Larvae Infection, Sampling and Lysis

WT AB zebrafish larvae were inoculated with CyHV-3 EGFP (1.2 × 10^6^ PFU/mL) or mock-infected with PBS via pericardial microinjection. The larvae were placed in 24-well plates with 1 mL E3 medium per well and incubated at 25 °C. Infected and mock-infected larvae were sampled at 1, 2 and 4 dpi (triplicates at each timepoint with 5 larvae pooled per replicate). Prior to sampling, larvae were euthanized using an overdose of buffered tricaine in E3 media (400 mg/L). Each replicate group of euthanized larvae was transferred immediately to 1.5 mL tubes, excess E3 medium was removed, and 700 μL QIAzol lysis reagent (Qiagen, Hilden, Germany) was added. Whole larvae were then completely homogenized in lysis reagent by passing the lysate through a 21 G needle 20 times using a 2 mL syringe. After homogenization, lysates were stored at −80 °C until RNA isolation.

#### 2.4.2. RNA Isolation, Library Construction and RNA Sequencing

Larvae lysates were thawed and incubated at room temperature for at least 5 min and 140 μL chloroform was added to each sample. Lysates were then vortexed for 15 s, incubated at room temperature for 3 min and centrifuged for 15 min at 12,000× *g* at 4 °C. After centrifugation, 240 μL of the aqueous layer was removed and 360 μL of 100% ethanol was added with immediate mixing by pipetting. Samples were then added to RNeasy spin columns, and RNA was isolated using an RNeasy Mini Kit (Qiagen, Hilden, Germany) with on-column DNase treatment. RNA was eluted in 100 μL RNase-free water using two 50 μL elution steps, and split into smaller aliquots for storage at −80 °C. For each sample, a single aliquot was used to check the quality of RNA using an Agilent Bioanalyzer, ensuring that RNA integrity (RIN) values were at least 9.5 before proceeding. Samples were used as input for barcoded RNA-Seq library preparation using the TruSeq Stranded mRNA kit (Illumina), and libraries were sequenced using the Illumina NextSeq 500 System.

#### 2.4.3. Bioinformatics Analysis

Sequence reads were aligned to the zebrafish reference genome GRCz11 (Ref Seq: GCF_000002035.6) in order to generate gene expression data. The data were used to identify differentially expressed genes (DEGs) in infected samples relative to non-infected samples (defined as those with false discovery rate (FDR) adjusted *p*-values < 0.05). DEGs were analysed further to identify functional relationships, and expression data were analysed to identify gene-sets that were significantly enriched in infected samples relative to non-infected samples. A non-abbreviated summary of the bioinformatic analysis conducted in this study is provided in Methods S2.

### 2.5. Mutant Zebrafish Experiments

#### 2.5.1. Generation of Mutant Zebrafish Strains Using CRISPR/Cas9

**Figure 1:**
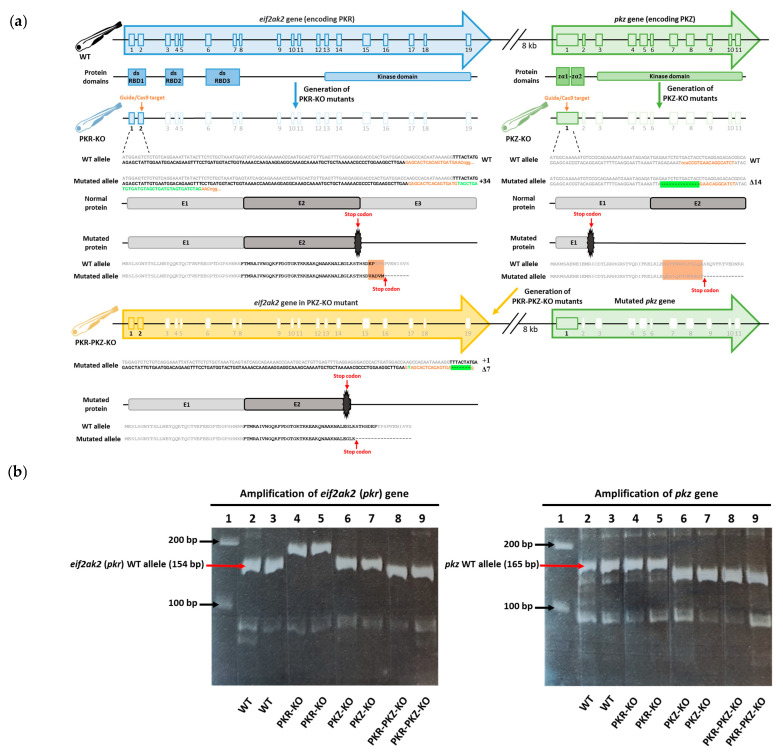
Generation and verification of CRISPR-Cas9 *eif2ak2* (*pkr*) and *pkz* mutations in zebrafish (**a**) Structure of zebrafish *eif2ak2* (*pkr*) and *pkz* genes and proteins. The protein domains including double stranded RNA-binding domains (dsRB), Z-DNA/RNA binding domains (zα) and kinase domains are aligned to the corresponding exons. The CRISPR/Cas9 gene editing targets were exon 2 in zebrafish *eif2ak2* gene and exon 1 in zebrafish *pkz* gene; sgRNA target sequences are also displayed (orange, PAM lower case). The CRISPR/Cas9-induced changes in the WT *eif2ak2* gene (34-base insertion) to generate PKR-KO, and WT *pkz* gene (14-base deletion) to generate the PKZ-KO mutant strains are displayed. After the generation of the PKZ-KO mutant strain, the WT *eif2ak2* gene in this strain was also mutated, resulting in the PKR-PKZ-KO mutant strain (displayed below). The mutated *eif2ak2* gene in the PKR-PKZ-KO strain exhibits a different mutation (7-base deletion with 1-base insertion) relative to the mutated *eif2ak2* gene in PKR-KO mutant. Inserted and deleted sequences are highlighted in green (deleted sequences are represented by “-“). (**b**) Results from genotyping of homozygous WT, PKR-KO, PKZ-KO and PKR-PKZ-KO zebrafish groups. This involved PCR amplification of *eif2ak2* (*pkr*) and *pkz* genes, in each mutant group (left and right gel images, respectively, with expected sizes of WT alleles indicated). Each gel consists of the same layout: Lane 1: 1kb Molecular Marker, Lanes 2–9 each represent a DNA extracted from single whole larva, Lanes 2–3: WT Larvae, Lanes 4–5: PKR-KO mutants, Lanes 6–7 PKZ-KO mutants, Lanes 8–9 PKR-PKZ-KO mutants. Mutant *eif2ak2* (*pkr*) alleles were detected in PKR-KO and PKR-PKZ-KO larvae exhibiting 188-bp and 148-bp amplicons, respectively (left gel). The mutant *pkz* allele was detected in in PKZ-KO and PKR-PKZ-KO larvae, both exhibiting 151-bp amplicons (right gel). Higher quality figures for the whole manuscript are available in the PDF version.

The *mut eif2ak2* (*pkr*)*^ulg025^*, *mut pkz^ulg027^*, and *mut eif2ak2* (*pkr*) *L15-1* knockout (KO) zebrafish lines, hereafter referred to as the PKR-KO, PKZ-KO and PKR-PKZ-KO mutant strains, were generated by CRISPR/Cas9 technology as described previously [54,55,56]. The nls-zCas9-nls mRNA was synthesized by transcription of the plasmid pT3TS-nCas9n (Addgene #46757). First, WT strain AB zebrafish were used to generate the mutant strains PKR-KO and PKZ-KO (Figure 1a). To generate the PKR-KO and the PKR-PKZ-KO mutant strains, CHOPCHOP [57] software was used to design two single-guide RNAs (sgRNA) GAGCACTCACAGTGATGAACCGG and CCACCGTGAACAGGCATCT (PAM motifs are underlined) to target exon 2 of the WT *eif2ak2* (or *pkr*) gene (NCBI/Entrez/GenBank Gene ID: 100001092) and exon 1 of the WT *pkz* gene (NCBI/Entrez/GenBank Gene ID: 503703), respectively (Figure 1a). sgRNAs were generated by in vitro transcription from oligonucleotide templates using the MEGAscript T7 transcription kit (Ambion) as described previously [58]. The DNA templates were prepared by annealing and filling two oligonucleotides containing the T7 promoter sequence and the target sequences as previously described [56]. One-cell stage zebrafish embryos were injected with approximately 1 nL of a solution containing 50 ng sgRNA and 300 ng nls-zCas9-nls mRNA. The efficiency of mutagenesis was checked by genotyping using heteroduplex migration assays after amplification of targeted genomic sequences. Founder embryos (F0 generation) carrying a germline mutation in *eif2ak2* or *pkz* were raised to adulthood and outcrossed with WT fish to generate heterozygous F1 fish. Fish harbouring frameshift mutations were kept and used to raise F2 homozygous stable knockout lines. Subsequently, PKZ-KO mutant strain zebrafish were then used to generate the double KO PKR-PKZ-KO mutant strain by repeating the process used to generate the PKR-KO mutant strain (Figure 1a). These mutations all resulted in genes producing truncated proteins and were verified by PCR (Figure 1b).

#### 2.5.2. Genotyping of Zebrafish Mutant Lines

The genotyping of WT, PKR-KO, PKZ-KO, and PKR-PKZ-KO zebrafish was performed by polymerase chain reaction (PCR). In order to extract the DNA, two randomly selected zebrafish larvae (4 dpf) were euthanized per mutant line. Each larva was transferred to an Eppendorf tube containing 25 µL 50 mM NaOH, heated at 95 °C for 25 min, and cooled on ice for 10 min. Finally, 2.5 µL 1M Tris-HCl pH8.0 was added, and cellular debris was pelleted by brief centrifugation for 15 sec. DNA concentration was determined by measuring A260 (NanoDrop 2000, Thermo Scientific, Waltham, NJ, USA), and ~2.5 µL of the resulting lysate was used per standard PCR reaction with gene-specific primers (Table S1). PCR reactions consisted of 1× Thermopol buffer (New England Biolabs, Ipswich, MA, USA), 0.025 U/µL Taq Polymerase (New England Biolabs), 300 nM forward and reverse primers, and 60 nM dNTPs (total volume 25 μL). The cycling conditions were as follows: 95 °C for 2 min, 40 cycles of 45 s at 95 °C, 45 s at 60 °C, 20 s at 72 °C, and ending with 72 °C for 10 min.

#### 2.5.3. Quantification of Viral Genome by TaqMan PCR

Larvae were euthanized using an overdose of tricaine, transferred into RNAlater (Thermo Fisher, Waltham, NJ, USA) and stored at −20 °C. DNA was extracted from whole larvae with a DNeasy Tissue Kit (Qiagen, Hilden, Germany), and approximately 1 ng genomic DNA was used for each TaqMan PCR reaction. TaqMan qPCR reactions consisted of 1× IQ Supermix (Bio-Rad, Hercules, CA, USA), 200 nM forward and reverse primers, and 400 nM TaqMan probe (total volume of 25 μL). The primers and probes used are provided in Table S1. The PCRs were performed using a CFX96 Touch real-time PCR detection system (Bio-Rad, Hercules, CA, USA) with detection in the FAM channel. The cycling conditions were as follows: 95 °C for 15 min, 40 cycles of 15 s at 94 °C, and 60 s at 60 °C. Each sample was analysed in triplicate. Viral genome copies were normalized to zebrafish genome copies (internal control) by also amplifying zebrafish genomic DNA as described previously [59]. Viral and zebrafish (internal control) PCRs were performed in separate wells, but always on the same plates. Negative template controls and positive controls were included on each plate. Data were exported to Excel using CFX Manager v3.0 software (Bio-Rad, Hercules, CA, USA). Relative levels of viral genome copies were calculated using the 2^−△△CT^ method as described previously [60].

### 2.6. Statistical Analysis

Each dataset was first tested for normality using the Shapiro–Wilk test, which was conducted as a stand-alone test or as part of a two-way ANOVA analysis of residuals implemented in GraphPad Prism (v8.0.1). The omnibus tests used were dependent on the outcome of the Shapiro-Wilk tests. For datasets exhibiting normal distribution, One-way ANOVA, Two-way ANOVA, or Two-way repeated measures (RM) ANOVA were used and implemented in GraphPad Prism. For datasets not exhibiting normal distribution, the Durbin test was used (PMCMR package v4.4 [61]), implemented in R (v4.2.0) [62]. The variables of interest relating to each of these tests and their significance are described in the text. Survival curves were compared using Logrank tests implemented in GraphPad Prism

Post-hoc multiple comparisons between groups of interest were made using either the Sidak test (two groups) or the Tukey test (more than two groups) implemented in Graphpad Prism (in conjunction with ANOVA tests), for data exhibiting normal distribution. Multiple comparisons were made using Dunn’s pairwise test (FSA package v0.9.3 [63]) with Benjamini-Hochberg *p*-value adjustment done using the p.adjust function in R (in conjunction with the Durbin Test), for datasets not exhibiting normal distribution. For the purposes of visual clarity, only significant results from post-hoc multiple comparisons are indicated in each corresponding figure. The results of multiple comparisons tests are represented using the following symbols, * *p* < 0.05; ** *p* < 0.01; *** *p* < 0.001; **** *p* < 0.0001.

## 3. Results and Discussion

### 3.1. ZF4 Cells Express Low Susceptibility and Reduced or Even No Permissivity to Cyprinivirus Infection Leading to Abortive Infection of Cell Monolayers

**Figure 2:**
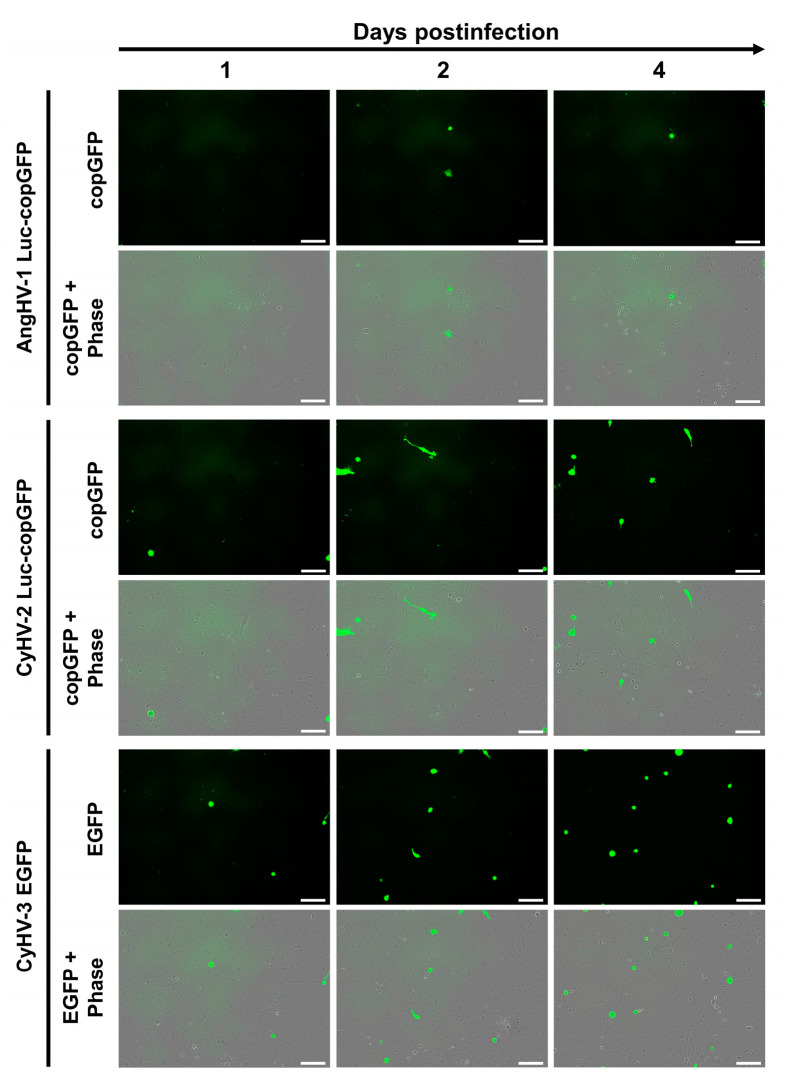
Infection of ZF4 cells by cypriniviruses. ZF4 cells were infected with the AngHV-1 Luc-copGFP, CyHV-2 Luc-copGFP and CyHV-3 EGFP recombinant strains. Infection progression was imaged by epifluorescence microscopy. Infected cells were identified based on green fluorescence expression at the indicated timepoints of infection. Scale bars = 100 µm.

In this experiment we tested the susceptibility and permissivity of the ZF4 cell line to infection with AngHV-1, CyHV-2 and CyHV-3, using recombinant strains expressing green fluorescent proteins as reporters. Cells were monitored from 1 dpi onwards using epifluorescence microscopy. At 1 dpi, infected cells were observed, with much less AngHV-1 infected cells relative to CyHV-2 and CyHV-3. The amount of CyHV-2 and CyHV-3-infected cells increased from 1–4 dpi, while the amount of AngHV-1-infected cells decreased after 2 dpi (Figure 2).

Syncytia formation, lysis plaques, or other cytopathic effects (CPE), were not observed in monolayers infected with CyHV-2 or CyHV-3. Together, these data revealed that ZF4 cells expressed some level of susceptibility to the cypriniviruses tested, no permissivity to AngHV-1 infection, and greatly reduced permissivity to CyHV-2 and CyHV-3 infection relative to typical observations in cells derived from their respective natural hosts that are routinely used for culture of these viruses.

**Figure 3:**
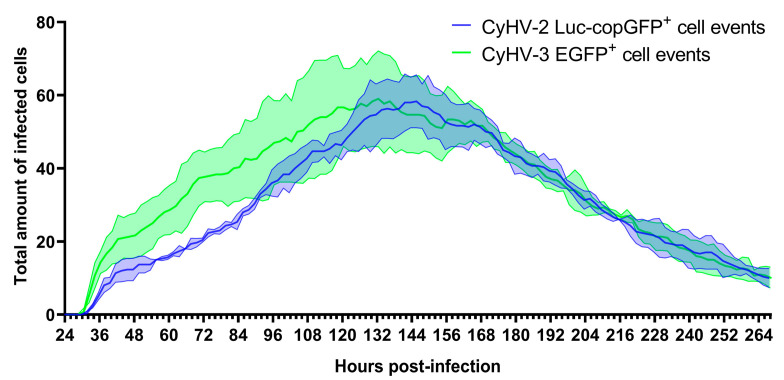
Quantification of CyHV-2 and CyHV-3-infected cells in ZF4 monolayer over time. This data was acquired via time-lapse fluorescent microscopy (IncuCyte). Cells were cultured in a 24-well plate and infected with CyHV-2 Luc-copGFP or CyHV-3 EGFP recombinants (1.2 × 10^6^ PFU/mL for each recombinant). At 24 hpi, cells were imaged every 2 h for 11 days. Data represent the mean ± standard errors from three replicates/wells. Data from each replicate at each timepoint represent the sum of fluorescent cells observed in nine separate locations of each well.

To further characterise the infection of ZF4 cells by the three cypriniviruses in a more quantitative manner, we utilized timelapse microscopy (Figure 3). ZF4 cells were infected, and the numbers of infected cells present with respect to time were tracked from 1–11 dpi as illustrated in Figure S2. Again, the number of AngHV-1 infected cells were low relative to CyHV-2 and CyHV-3 infections and did not increase over time. Consequently, AngHV-1 was excluded from further quantification analysis in vitro. None of the infections led to the formation of detectible CPE. We observed a steady increase in CyHV-2- and CyHV-3-infected cells from ~24–144 hpi, followed by a rapid clearance of both viruses from ZF4 monolayers (Figure 3). As evident in Figure 2, during the most rapid period of virus propagation within the monolayer (from ~24–144 hpi) the rate of CyHV-2 and CyHV-3 spread was not exponential (Figure 3), indicating poor replication efficiency within infected cells and/or reduced transmission of progeny virus to additional cells.

In the CyHV-2-infected monolayers, the peak of infected cells occurred at 146 ± 4 hpi with a mean of 58 ± 7 infected cells observed per well (sum of nine different fields of view in each well, sums from three replicate wells used to derive mean). This peak occurred earlier in CyHV-3-infected monolayers at 124 ± 11 hpi with a mean of 59 ± 13 infected cells at this point. Overall, time postinfection was shown to have a significant effect on the number of CyHV-2 and CyHV-3-infected cells observed (Two-way RM ANOVA, *p* value < 0.0001), but there was no significant difference between the two viruses in this respect (Two-way RM ANOVA, *p* value = 0.3164) (Figure 3).

However, from ~24–144 hpi, the mean number of infected cells tended to be higher in the CyHV-3-infected monolayer. For example, at 48 hpi there was a mean of 21 ± 6 CyHV-3-infected cells observed per well compared to a mean of 12 ± 3 CyHV-2-infected cells. From ~144–264 hpi, the number of infected cells evolved similarly for both viruses, with infected cell numbers decreasing steadily until the end of the experiment, representing the gradual clearance of infected cells from the monolayer (Figure 3). Notably this clearance was largely characterized by apoptosis-like morphological changes.

**Figure 4:**
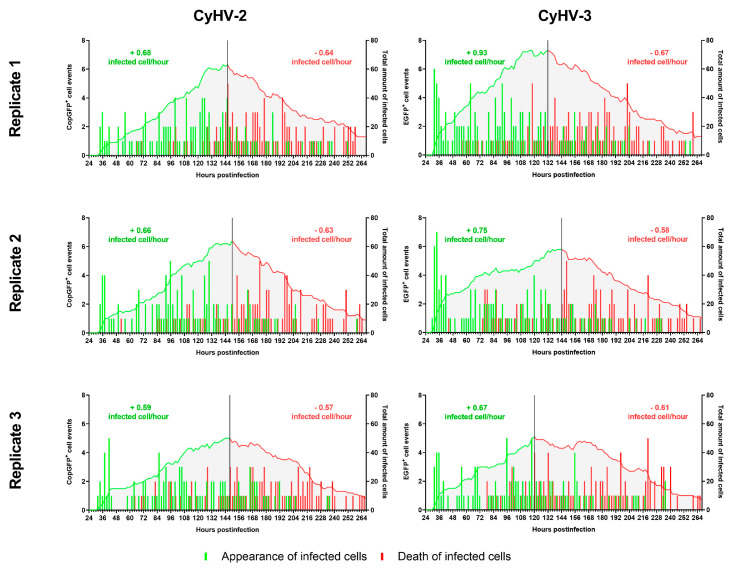
Kinetics of appearance and death of CyHV-2 and CyHV-3-infected cells before and after infection peak. The bars relate to the temporal pattern of appearance and disappearance of CyHV-2-infected or CyHV-3-infected cells (based on fluorescent reporter expression). The quantities are based on the total amount of observations made in 9 different locations in each well/replicate. The green and red curves show the total amount of infected cells up until the peak of infection (represented by the black vertical line) and after the peak, respectively. The values on top of the curves represent the average rate of appearance of infected cells per hour (green) and the average rate of death per hour (red). Analysing the rate of appearance/hour before the peak for CyHV-2 and CyHV-3 revealed no differences between the viruses.

These two time-ranges, i.e., ~24–144 hpi and ~144–264 hpi, corresponded to periods approximately before and after the peak of infected cells, respectively. Thus, we further scrutinized these two distinct periods separately in order to determine the extent of any differences between CyHV-2 and CyHV-3. After defining the timepoints corresponding to the latest infection peak in each replicate, we examined the two distinct periods of infection, comprised of viral propagation (pre-peak) and clearance (post-peak), by quantifying the appearance (beginning of infection) and disappearance (cell death) of infected cells (Figure 4). This revealed that infected cells appeared at a mean rate of 0.64 ± 0.05 cells per hour for CyHV-2 and 0.78 ± 0.13 cells per hour for CyHV-3 before the peak, with no significant differences between the two viruses in this respect (Two-way ANOVA, *p* value = 0.1704). It also revealed that a mean of 75 ± 4.16% and 72 ± 8.89% of newly infected CyHV-2 and CyHV-3 cells appeared before the peak of infection, respectively, in what appears to have been several waves of infection (Figure 4). For both viruses, in all replicates, an initial peak of infected cell appearance occurred at ~36 hpi, followed by a period of particularly low appearance of newly infected cells until after ~48 hpi. This may represent the transmission of the first generation of viral progeny to the second generation of infected cells (from ~24–36 hpi), and subsequent progeny to the next generation of infected cells (occurring after ~48 hpi). However, the low numbers of newly infected cells yielded from this transmission provides more evidence to support the possible inefficient replication and/or transmission of CyHV-2 and CyHV-3 between ZF4 cells (as observed in Figure 2).

For both CyHV-2 and CyHV-3, a substantial amount of newly infected cells (25–30%) appeared after the peak, even beyond 10 dpi, indicating that transmission of viral progeny was sustained into the later stages of the experiment. We have recently demonstrated that CyHV-3 virions lose infectivity rapidly in cell culture media (>95% by 24 h) [64], thus excluding the possibility that newly infected cells, particularly beyond 10 dpi, could have originated from the initial inoculum due to delayed viral entry into cells. It is also unlikely that we are observing delayed expression of viral genes, as all fluorescent reporters used in this experiment were driven by highly active constitutive promoters (CMV and EF-1α). Also, we reasoned that because cells expressing fluorescent reporters are actively cleared at increased rates and appear at decreased rates as the experiment continued, the outcome is distinct from that of spurious reporter expression (i.e., without expression of other viral genes, owing to integration of the expression cassette into the host cell genome), which should persist for longer without triggering cell death. Together, these observations indicated the occurrence of at least some viral progeny transmission to non-infected cells after an initial round of viral replication. However, as increase in the numbers of newly infected cells was not exponential, but linear, it indicated that efficient replication and/or transmission of CyHV-2 and CyHV-3 was very rare in ZF4 cells. Nonetheless, it provided evidence that ZF4 cells are transiently permissive to CyHV-2 and CyHV-3 infection. The observation of isolated infected cells without plaque formation indicated the absence of transmission via cell-cell contact. This may indicate a high degree of heterogeneity within ZF4 monolayers regarding susceptibility to these viruses, or very strong or fast innate responses in neighbouring cells. Within at least one permissive cell line, CyHV-3 cell-cell transmission may be greatly enhanced by syncytia formation (in particular with CyHV-3 FL strain derived recombinants, which we recently described [64]). However, we observed a notable lack of syncytia formation among CyHV-3-infected ZF4s, which may also contribute to reduced transmission via cell-cell contact.

It is important to note that for all viruses used in this study, the use of a high MOI of 3 (although calculated in the context of permissive cell lines used for viral production), did not result in many initial infected ZF4 cells, indicating a general lack of cyprinivirus susceptibility among ZF4 populations. This may happen for many reasons, for example, a lack of optimum cell surface receptors, resulting in inefficient viral entry. Conversely, entry may occur, but the viral replication may not commence due to a lack of crucial cellular factors. The exact reasons for this remain speculative and are beyond the scope of this present study, but it provides an opportunity for further investigation via single cell sequencing analysis in the future.

Notably, cell death before the infection peak was low with 74 ± 0.03% and 74 ± 0.07% of CyHV-2 and CyHV-3-infected cells dying after the peak, respectively. Therefore, the higher transmission, prior to the peak, was not reliant on the release of virions via infected cell lysis/death but rather on the normal mechanism of herpesvirus egress [65,66]. Programmed cell death prior to completion of the viral replication cycle in particular acts as an innate defence mechanism which infected cells can employ to reduce virus replication [67]. Indeed, this is what was observed post infection peak, with an increase in cell death correlating with a reduction in newly infected cells (Figure 4). We propose that relative to cells at the earlier stages of the experiment, both infected and uninfected cells present at later stages would have been subject to cytokine stimulation as part of the innate immune response. Even if such stimulation was transient, these cells (many of which may exhibit limited susceptibility to begin with) may have adopted a stronger antiviral-state at later stages of the experiment.

**Figure 5:**
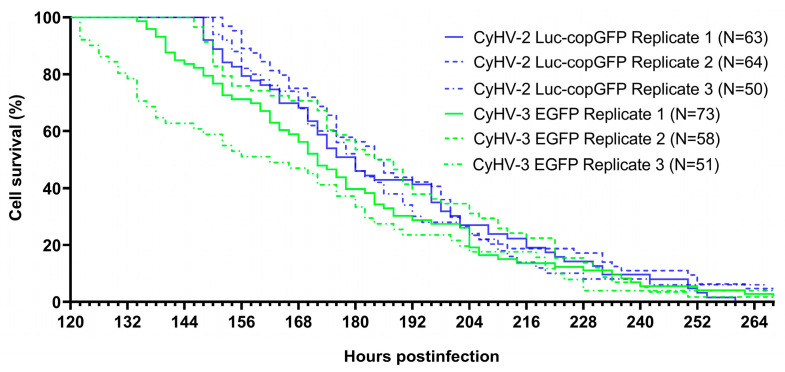
Survival kinetics for CyHV-2 and CyHV-3-infected cells displayed as Kaplan-Meier plots. CyHV-2 and CyHV-3-infected cells observed at 120 hpi were monitored until the end of the experiment. Cell death events and times were identified based on the disappearance of fluorescent signals (Figure S2). N = Number of cells followed.

In order to compare the virulence of CyHV-2 and CyHV-3 in ZF4 cells, we returned to the data Figure 3 and monitored all positive cells present at 120 hpi until their death, using this information to generate survival curves (Figure 5). This 120 hpi timepoint was selected, as it represented the earliest peak of infection out of the six that were defined in Figure 4, thus maximizing infected cell sample size while using a common timepoint for all groups. The median survival time for infected cells was 61 ± 3 h and 53 ± 12 for CyHV-2 and CyHV-3-infected groups, respectively. Although CyHV-2-infected cells tended to survive longer, there was no significant difference survival between the two groups (Log-rank Mantel-Cox test, *p* value = 0.0822).

**Figure 6:**
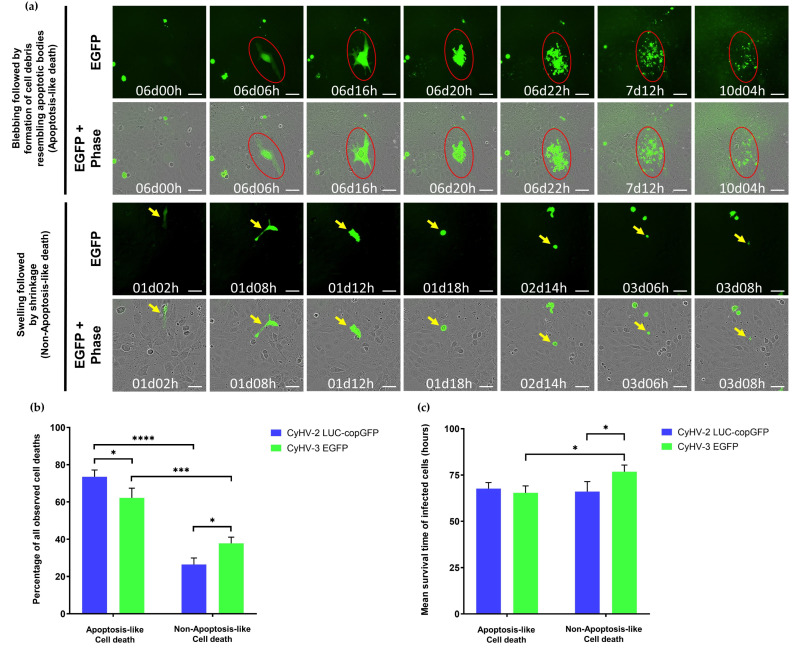
Cell death characteristics observed in CyHV-2 and CyHV-3 infections (**a**) Representative morphological observations among populations of infected cells (those exhibiting fluorescence) in the periods leading up to cell death (disappearance of fluorescence). Top panel: Morphological features consistent with apoptosis (cell shrinkage, membrane blebbing followed by the appearance of cell debris resembling apoptotic bodies, and progressive decrease of fluorescent signal). Bottom panel: Morphological features not consistent with apoptosis (cell swelling, followed by cell shrinkage, and absence of cell debris resembling apoptotic bodies prior to disappearance of fluorescent signal). Key examples of individual cells undergoing apoptosis-like and non-apoptosis-like death in each panel are highlighted by red circle and yellow arrows, respectively, which track the progression of morphology in a single cell with respect to time. Time postinfection (in days and hours) is indicated in images. Scale bars = 100 µm. (**b**) Percentage of infected cells exhibiting features of apoptosis-like or non-apoptosis-like cell death among those that died during the observation period (**c**) Mean survival time of infected cells undergoing cell death during the observation period according to the type of death observed. Data represents mean ± standard error from 3 replicates. **** *p* < 0.0001; *** *p* < 0.001; * *p* < 0.05.

In the majority of cases, death events were morphologically consistent with apoptosis, i.e., cell shrinkage, membrane blebbing leading to the appearance of cell debris resembling apoptotic bodies [68,69,70]) (Figure 6a, top panel). However, the occurrence of apoptosis was not definitively confirmed by staining. We also observed another distinct type of cell death that was not morphologically consistent with apoptosis. In these cases, morphological features mostly included initial cell swelling, followed by cell shrinkage and an absence of cell debris resembling apoptotic bodies prior to disappearance of fluorescent signal (Figure 6a, bottom panel). This is somewhat morphologically consistent with necrosis, where cell-death is associated with membrane rupture and leaking of cytoplasmic contents [68,69,70].

Notably, necrosis can also be initiated in a highly regulated manner known as necroptosis, which acts as a back-up for apoptosis [71,72]. However, it was not possible to differentiate between necrosis and necroptosis based on our morphological observations alone, and as with apoptosis, neither were definitively confirmed via staining. In any case, the apoptosis-like form of cell death was observed to be the dominant form of death among infected cells (Figure 6b). However, there were differences between the two viruses in this respect (Two-way ANOVA, *p*-value = <0.0001), with the proportion of CyHV-3-infected cells undergoing apoptosis-like cell death being significantly lower (Figure 6b), possibly indicating that CyHV-3 may be more efficient at blocking this apoptosis-like death in ZF4 cells. There was no significant difference between the two viruses in terms of survival times (Two-way ANOVA, *p*-value = 0.1112). However, among CyHV-3-infected cells, those undergoing non-apoptosis-like death exhibited significantly longer survival times than those undergoing apoptosis-like death (Figure 6c).

Previously, a separate study demonstrated that CyHV-3 could indeed infect ZF4 cells, with increasing viral RNA levels observed from 1–4 dpi, and an absence of CPE was also noted [38]. However, the viral dosages used were not directly comparable with this present study, and the possibility of viral clearance after 4 dpi was not investigated. In this present study, we monitored the progression of CyHV-3 infections for much longer (up to 11 dpi). Crucially, through the exploitation of reporter genes, in addition to demonstrating viral gene expression, we were also able to identify and quantify new cell infection events. This revealed continuous CyHV-3 transmission right up until the clearance of infection, albeit with increasingly reduced rates of newly infected cells. While we demonstrated that ZF4s are certainly susceptible to CyHV-3 infection, any initial productive infections leading to transmission of viable progeny were not sustained. Thus, ZF4 cells are transiently permissive to CyHV-3 with inefficient viral replication/transmission unable to overcome the innate immune response among infected and non-infected cells. This may be similar to previous observations with snakehead fish vesiculovirus (SHVV) infections in ZF4 cells where initial increases in virus levels were followed by a decrease, corresponding to ISG upregulation [73].

Unlike CyHV-3, prior to this study, the susceptibility ZF4 cells to AngHV-1 and CyHV-2 had not been investigated. Our results indicate that while ZF4 cells are also susceptible to both AngHV-1 and CyHV-2 infection, they are only permissive to the latter. However, as with CyHV-3, permissiveness to CyHV-2 infection was moderate and transient. These similarities between CyHV-2 and CyHV-3, and their differences to AngHV-1 in this context may reflect the fact that CyHV-2 and CyHV-3 are phylogenetically closer to each other, than each are to AngHV-1 [40,74,75,76]. Furthermore, given their natural host species, it stands to reason that CyHV-2 and CyHV-3 may also be inherently better adapted to growing in ZF4 cells relative to AngHV-1. Despite the lack of sustained permissivity to cypriniviruses, these in vitro experiments with ZF4 cells did provide some indication that the same recombinant viruses may be used to study transient cyprinivirus infection and clearance in zebrafish larvae, which, for many reasons (outlined earlier), may represent a valuable virus-host model.

### 3.2. Zebrafish Larvae Are Susceptible to CyHV-2 and CyHV-3 but Not to AngHV-1 Infection. Inoculation by the Two Cyprinid Herpesviruses Leads to an Abortive Infection

**Figure 7:**
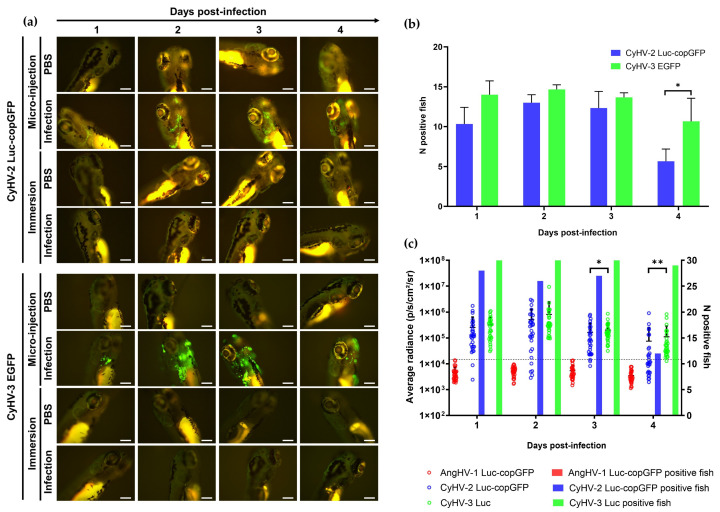
Susceptibility and permissivity of zebrafish larvae to infection with cypriniviruses after inoculation by microinjection (**a**) Epifluorescence microscopy images representative of larvae inoculated with CyHV-2 and CyHV-3 according to time postinfection (longitudinal observation of the same larvae over all timepoints) Scale bars = 200 µm. (**b**) Numbers of CyHV-2 and CyHV-3-infected larvae among groups inoculated by microinjection (n = 15). Data represents mean ± standard errors from 3 independent experiments (longitudinal observation of the same larvae over all timepoints). (**c**) Levels of AngHV-1, CyHV-2 and CyHV-3 detected in infected larvae according to time postinfection based on Luc2 signal expressed by viral recombinants. The data points represent the mean radiance per larvae according to time postinfection with mean ± standard error represented for each group at each timepoint (n = 30). The discontinuous line represents the cut-off for positivity and represents the mean + 3 × SD of the values obtained for mock-infected larvae. The number of positive larvae at each timepoint is represented by bars. * *p* < 0.05; ** *p* < 0.01.

We next investigated the susceptibility and permissivity of zebrafish larvae to the same three cypriniviruses. To investigate this, we used WT AB zebrafish larvae at 3 dpf. In the first experiment, larvae were infected with the same recombinants previously used (Figure 2, Figure 3, Figure 4, Figure 5 and Figure 6). Larvae were inoculated by pericardial microinjection with 1.2 × 10^6^ PFU/mL of each recombinant or PBS. In parallel, larvae were also infected by immersion with a final concentration of 4000 PFU/mL of each recombinant or PBS. The susceptibility of larvae to these viruses was assessed using epifluorescence microscopy to detect reporter expression from each recombinant. Independently of the mode of inoculation used or the virus, no morbidity or mortality was observed among larvae. Epifluorescence microscopy indicated no infection in larvae inoculated by immersion. Conversely, viral infection was detected from 1 dpi in larvae inoculated with CyHV-2 and CyHV-3 by microinjection (Figure 7a) with no fluorescence detected in the AngHV-1 inoculated group. Fluorescence intensity in CyHV-2 and CyHV-3-infected larvae increased from 1–2 dpi. However, as per earlier in vitro observations, these infections were transient, with fluorescence intensity (Figure 7a) and the numbers of infected larvae (Figure 7b) decreasing by 4 dpi. While the pattern was similar for both viruses, the CyHV-3 group exhibited greater fluorescence intensity and significantly higher rates of infected larvae (Two-way RM ANOVA *p*-value = 0.0214). Infection clearance was most pronounced in the CyHV-2-infected group, with a significantly higher proportion of larvae infected at earlier timepoints exhibiting viral clearance by 4 dpi relative to CyHV-3 (Figure 7b). The differences between these three cypriniviruses were investigated further by measuring Luc2 expression from recombinants, representing a more quantitative comparison of viral levels in vivo. This involved the same AngHV-1 and CyHV-2 recombinants used in Figure 7a, with CyHV-3 EGFP replaced with CyHV-3 Luc. Larvae were inoculated or mock-inoculated as per Figure 7a. Again, no mortality was observed in any groups and no infection was detected in the AngHV-1 group. The CyHV-3-infected group exhibited significantly higher viral levels relative to CyHV-2 (Durbin Test, *p*-value = 0.0008), indicating that CyHV-3 replicates better in this model. Also, for both CyHV-2 and CyVH-3, a reduction in virus levels occurred at 3 dpi, coinciding with a reduction in the numbers of infected fish, indicating the initiation of viral clearance. However, as per Figure 7b, clearance was significantly greater within the CyHV-2-infected group by 4 dpi (Figure 7c).

These experiments revealed that zebrafish larvae are not susceptible to any of these viruses via immersion, which may be considered a more natural route. This is similar to previous findings with CyHV-3 in Tübingen zebrafish larvae [38]. Conversely, larvae were susceptible to CyHV-2 and CyHV-3 when inoculated via pericardial microinjection, but not to AngHV-1 via the same route. In line with earlier observations in vitro, CyHV-2 and CyHV-3, which naturally infect members of the family *Cyprinidae*, are much more fit in this zebrafish model relative to AngHV-1. For CyHV-2 and CyHV-3, a peak of infection was reached at 2 dpi, with viral clearance initiating from 2–3 dpi. Notably, this is the first report of cyprinivirus infection in zebrafish larvae. Our observations are largely consistent with previous description of CyHV-3 infections in adult zebrafish (inoculation by intraperitoneal injection) [38]. There was also a notable lack of mortality in previous studies involving the challenge of zebrafish with other viruses of cyprinid fish [38]. One explanation is that zebrafish may naturally possess robust defences against other viruses that are closely related to CyHV-2 and CyHV-3 which may have circulated in their natural habitat during their evolution. However, few viruses are known to naturally infect zebrafish [1,19], thus it would be useful to determine if any extant uncharacterized members of the family *Alloherpesviridae* naturally infect zebrafish as a primary host, as it would open up new avenues of investigation with a valuable homologous herpesvirus-host model in zebrafish. It is also possible that this lack of mortality is related to the viral dose or even inoculation site, both of which can impact the severity of viral infections in zebrafish larvae, as exemplified elsewhere [77,78].

Our observations indicated that CyHV-3 exhibits greater fitness in these zebrafish models relative to CyHV-2. Thus, in addition to CyHV-3 being the most studied and the archetype fish alloherpesvirus [39], it also represented a more valuable model to utilize in the further study of alloherpesvirus infections in zebrafish larvae. Thus, CyHV-3 was selected for all further in vivo investigations in this study.

### 3.3. Pericardial Inoculation of Zebrafish Larvae with CyHV-3 Leads to Infection of Resident and Motile Cells around the Inoculation Site Followed by Their Apoptosis-like Death and Viral Clearance

Earlier experiments revealed that the levels of CyHV-3 signal increased from 1–2 dpi with clearance commencing from 2–3 dpi (Figure 7a,c). However, it remained unclear if increases in viral signal were merely due to increasing levels of viral gene expression or the numbers of infected cells. We chose to investigate this using light sheet microscopy to capture epifluorescence and brightfield images at regular intervals in live CyHV-3-infected larvae from 2–3 dpi and subsequently generated a timelapse video with this data (Video S1). This timepoint was selected as it overlapped with the highest viral signals and the beginning of the viral clearance process (Figure 7), and because no viable virus from the original inoculum should have persisted to this timepoint [64].

**Figure 8:**
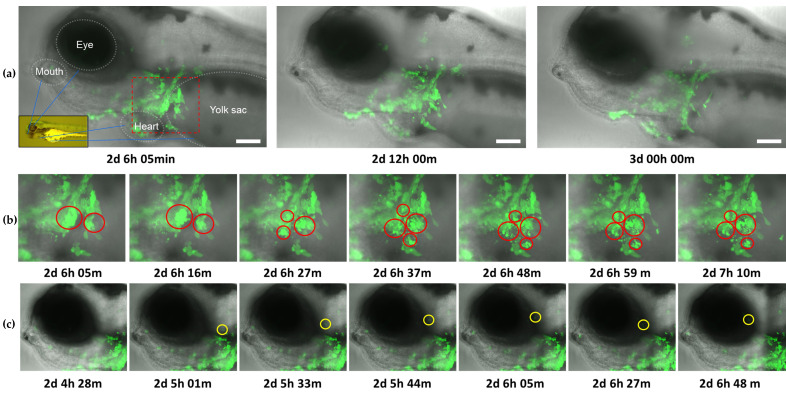
Frames from timelapse video of CyHV-3 EGFP infection in zebrafish larvae from 2–3 dpi (Video S1). The video represents overlay of brightfield/transmission and EGFP fluorescence (green). Time postinfection (in days, hours, and minutes) is indicated under each frame. (**a**) Entire field of view from light-sheet microscopy. For the purposes of visual orientation, identifiable anatomical features and corresponding locations within larvae body (inset image) are indicated in the first panel. Images show that the infection is primarily localized around the inoculation site (red square), and a decrease in viral levels from 2.5–3 dpi. Scale bars = 100 µm. (**b**) Enlarged images of the area within red square in (**a**), representing key examples of apoptosis-like death occurring among large numbers of infected cells (red circles) around the inoculation site, with such events primarily characterized by blebbing followed by the appearance of cell debris resembling apoptotic bodies (**c**) Key example of highly motile infected cell (highlighted with yellow circle), migrating away from the site of inoculation.

As per Figure 7a, the infection was mainly localized around the heart area, reflecting the inoculation route. In line with earlier observations, a reduction in viral levels commenced between 2.5–3 dpi (Figure 8a and Video S1). Notably, the data revealed a substantial upsurge in apoptosis-like cell death immediately prior to clearance, indicating that programmed cell death may also play a major role in this process in vivo (Figure 8b and Video S1). Although the occurrence of apoptosis in response to CyHV-3 infection in vivo was not confirmed by staining in this present study, our observations are similar to previous studies involving timelapse analysis of CHIKV-infected zebrafish larvae [23]. Throughout the monitoring period, highly motile cells, possibly macrophages or neutrophils, were also observed to be infected. These did not remain localized around the inoculation site. However, they were not observed to establish secondary infection sites elsewhere (Figure 8c and Video S1). Furthermore, some of these motile cells appeared also to undergo apoptosis-like and non-apoptosis-like cell death consistent with necroptosis (Video S1). Unlike earlier in vitro observations, this data did not provide unambiguous evidence of newly infected cells appearing before clearance commenced. Indeed, the induction of a programmed cell death response among infected cells in vivo, thus interrupting the CyHV-3 replication cycle, would lead to a reduction in successful CyHV-3 transmission to new cells. Consequently, CyHV-3 propagation in vivo may be sufficiently restricted to facilitate its clearance via the innate immune response alone. This hypothesis still implies that zebrafish cells are inherently permissive to CyHV-3 replication. However, this would, at the very least, require expression of all essential CyHV-3 protein coding genes in vivo. Thus, we subsequently investigated this and the nature of the innate immune response via transcriptomic analysis of infected larvae.

### 3.4. Transcriptomic Analysis of Infected Zebrafish Indicate Upregulation of ISGs, in Particular Those Involved in Programmed Cell Death, Innate Immune Response and PRR Signalling Pathways

In order to further characterise the response to CyHV-3 infection in this zebrafish larvae model in terms of the ISG upregulation, the potential involvement of programmed cell death (as indicated in Figure 8 and Video S1), and to establish the extent of CyHV-3 gene transcription in this model, we conducted transcriptomic analysis of infected zebrafish larvae. CyHV-3-infected and mock-infected larvae were sampled at 1, 2, and 4 dpi for RNA extraction and sequencing. RNA-Seq, yielded ~15–20 million reads per sample with data publicly available under BioProject Accession number PRJNA929940. Gene expression was compared between infected and mock-infected samples at each timepoint to identify DEGs. In line with viral levels observed in earlier experiments, viral RNA levels reached a peak at 2 dpi (0.34% of total transcriptome), falling considerably by 4 dpi (Table S2). Notably, transcription from all 155 CyHV-3 ORFs was detected by 2 dpi (Figure S3 and Table S3), indicating that indeed, in this model, cells may be permissive to CyHV-3 replication. Host differential gene expression in response to infection also peaked at 2 dpi, with 7.4% of expressed genes classified as DEGs (Table S2 and Figure S4).

Prior to this study, it was unknown how zebrafish larvae respond to CyHV-3 challenge in terms of type I IFN gene expression. Consistent with other reports [16], we found that *ifnphi2* was not expressed at this developmental stage. The IFN response in zebrafish larvae relies on expression of *ifnphi1* and/or *ifnphi3* genes [16]. However, we did not observe convincing expression from either gene at any timepoint. Our sampling points range from at 1–4 dpi, which equate to 96–168 hpf, with previous studies indicating that WT AB zebrafish larvae are capable of expressing *infphi1* and *infphi3* by this developmental stage [16,23]. Notably, these previous studies, involving SVCV and CHIKV challenge, utilized RT-qPCR to detect IFN gene transcription, which may be more sensitive than RNA-Seq in some situations.

While CyHV-3 is known to inhibit the IFN-response in vitro [79,80], our observations do not necessarily indicate inhibition of the IFN-response in zebrafish. It is possible that the upregulation of IFN genes occurs very early after infection, returning to basal levels rapidly, prior to the first sampling point. The effects of this rapid and short-lived IFN response should be still observed in the form of subsequent ISG induction. Indeed, in this present study, the list of the 250 most significant DEGs at 2 dpi is dominated by typical ISGs (Table S4). This ISG induction in the absence of IFN detection is similar to previous studies with WT zebrafish larvae infected with nervous necrosis virus (NNV) [78]. In both studies, it is likely that IFN upregulation occurred prior to the earliest sampling point. However, the kinetics of Type I IFN induction in WT AB zebrafish may depend on the nature of the viral challenge (virus, dosage and inoculation site/route). For example, in previous studies in which WT AB larvae were inoculated with HSV-I and CHIKV (72 hpf), *ifnphi1* upregulation peaked at 36 hpi [77] and 24hpi [23], respectively, with further differences in sustained upregulation after these timepoints. Furthermore, the expression of *infphi1* and *ifnphi3* may be model-specific. For example, Tübingen strain zebrafish larvae inoculated with Tilapia Lake Virus (TiLV) (48–60 hpf) were only observed to exhibit significant *ifnphi1* upregulation but not insignificant *infphi3* upregulation by 48 hpi [81]. It remains unclear if only one or both IFN genes are responsible for this ISG induction (Table S4) in our infection model, and this will be the subject of future studies, involving sampling at earlier timepoints.

**Figure 9:**
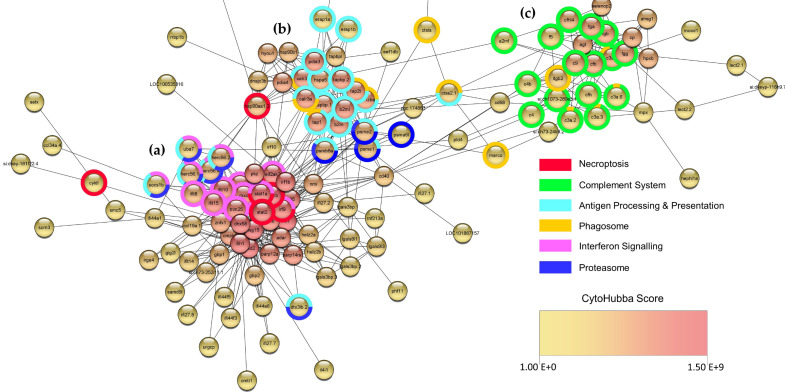
Network representing the functional associations between some of the top 250 most significant DEGs at 2 dpi. Using STRING protein query function in Cytoscape, 208 of the top 250 most significant DEGs were identified and scored based on functional association with each other. These data were used to generate a network in Cytoscape, which was then arranged based on GeneMania force directed layout. Each DEG is represented by a node, with edges (connecting lines) representing functional association. The largest contiguous network resulting from this analysis (136 nodes and 696 edges) is displayed. For visualization purposes, nodes in the peripheral regions of the network (representing DEGs *LOC100006895*, *rnasel3*, *ndrg1b*, *pde6ha*, and *serpinb1l1*) were omitted. This resulted in one large cluster (**a**), and two smaller clusters (**b**) and (**c**). STRING functional enrichment analysis indicated that most DEGs in this network were related to the immune response to infection (Table S6), and genes were labelled based on the main types of gene-set categories enriched in each of their respective clusters. This revealed distinct functions associated with each gene cluster, for example (**a**) interferon and PRR signalling, (**b**) antigen processing and presentation, and (**c**) complement response. The network was also analysed by CytoHubba, which was used to identify the potentially most important hub nodes within the network, with each node scored and coloured based on maximal clique centrality within the network, according to the CytoHubba score colour scale provided; however, this is better represented in Figure S5, with corresponding CytoHubba scores in Table S7.

We also conducted further characterisation of the main types of genes that were differentially expressed in response to CyHV-3 infection in zebrafish larvae. Using STRING, we generated a network (Figure 9) representing the functional relationships between the top 250 most significant DEGs at 2 dpi (Table S4). As expected, functional enrichment analysis of this network revealed that these DEGs were mainly associated with the immune and stress responses (Table S6). Three main clusters formed within this network. The largest cluster (Figure 9a) mainly represented genes involved in viral infection and cytokine responses. These include genes encoding the antiviral GTPase proteins such as *mxa*, *mxb*, *mxc*, and *mxe*, as well as *rsad2* (or *vig-1*, *viperin*). This is consistent with previous observations in zebrafish larvae infected with NNV [78], Zebrafish Picornavirus (Zfpv) [19], and CyHV-3-infected adult zebrafish [38]. In terms of the cytokine response, genes encoding IFN regulatory factors *irf7* and *irf9* were also part of this main cluster. Notably, zebrafish *irf3* was also among the top 250 most significant DEGs (Table S4), however as STRING returned no results for this gene, it was not included in the network in Figure 9. In addition, genes encoding other important elements of the IFN response, *stat1a*, *stat1b, stat2*, and augmentation and regulation of this response such as *isg15* [30] were also featured in this cluster, consistent with zebrafish larvae responses to HHV-1 [77] and NNV [78].

The detection of “non-self” material in cells via PRRs is an important part of the innate immune response. Viral nucleic acids represent major PAMPs during infections, and genes encoding PRRs to detect these PAMPs were among the most significant DEGs in our experimental model. For example, genes encoding important zebrafish RIG-I-like receptor (RLR) orthologs, such as *ifih1* (encoding *MDA-5* ortholog) [82], and *dhx58* (encoding *LGP2* ortholog) [83] were centrally located within this large cluster (Figure 9a). An additional gene, *rigi*, encoding the zebrafish ortholog of RIG-I, the most-studied RLR [84], was also significantly upregulated in response to infection, but not among the top 250 most significant DEGs used to generate this network (274th most significant DEG, Table S5). Genes encoding other important components of the RLR viral RNA sensing apparatus such as *trim25* [85,86] were also centrally located in this large cluster (Figure 9a). In addition to RLRs, other genes encoding RNA binding proteins are important actors in the innate immune response such as *adar* [87], *eif2ak2* (encoding PKR ortholog) [88], *pkz* [88,89,90,91], and *ifit10* (human *IFIT5* ortholog) [92,93,94] also co-locate within the same large cluster. Interestingly, we noted that two additional genes, *helz2*a and *helz2*b, encoding proteins that may act as evolutionarily conserved RNA sensors [95], can be observed at the peripheral regions of this main cluster. Many known vertebrate dsDNA sensing PRRs are absent in teleost fish [95,96]. Of the few known genes encoding dsDNA sensing PRRs in zebrafish, which include *ddx41* [77,97], *cgasa* [98], *dhx9* [77], and *dhx36* (the latter of which, may act as a conserved RNA and DNA PRR [99]), only *cgasa* was significantly upregulated, but not featured in the top 250 DEGs (623rd most significant DEG, Table S5). This may indicate that RNA sensing as opposed to DNA sensing PRRs represent an important part of the response to CyHV-3 infection in zebrafish larvae, even though it is a dsDNA virus. This is consistent with growing evidence for the role of RLRs in the detection of dsDNA viruses, such as members of the family *Herpesviridae* or *Adenoviridae* [100,101,102,103,104].

Within the largest cluster, in addition to genes being generally involved in antiviral responses, functional enrichment analysis identified a subset of clusters representing genes belonging to IFN signalling and necroptosis gene-sets (Figure 9a). The same functional enrichment analysis indicated that genes in the smaller central cluster were mainly involved in antigen processing and phagosome responses (Figure 9b), with genes in the smaller cluster on the right mainly related to the complement system (Figure 9c). Furthermore, the identification of the potentially most important hub nodes within the network in Figure 9 (based on maximal clique centrality) revealed that nodes representing RNA PRRs *ifih1* (MDA5 ortholog) and *dhx58* (LGP2 ortholog) were ranked highest, along with *rsad2* (or *vig-1*, *viperin* ortholog), *stat1a*, *irf7*, *isg15* and *stat1b* (Figure S5 and Table S7). Notably, all the top ten ranked hub nodes (twenty in total) represent genes located in the largest cluster (Figure 9a), most of which are described above.

Interestingly, in addition to many commonly studied ISGs, we also observed upregulation of genes encoding NACHT-domain and leucine-rich-repeat-containing (NLR) proteins, for example, *loc100535428* (Table S4). These represent a protein-class that is now increasingly recognised as representing important elements of the innate immune response in teleost fish [19,105]. We also note the upregulation of many genes encoding uncharacterized products in response to CyHV-3 infection, some of which were >1000–5000-fold upregulated (Table S4). Focusing on those within the top 250 significant DEGs that were >100 fold upregulated, we noted that four of these were not previously described as being upregulated in response to infection or immune stimulation (Table S4). We also noted the upregulation of five non-coding RNA genes in response to CyHV-3 infection, one of which was >3000 fold upregulated (Table S4), representing the 6th most upregulated gene in the dataset. All other uncharacterized genes occurring within the group of top 250 most significant DEGs were further cross-referenced with existing GenBank entry information on predicted protein domains (Table S4). This revealed that three of these genes potentially encode additional NLR proteins, three encode RNA binding domains, and three encode proteins containing retrotransposon derived reverse transcriptase-like (RT-like) domains (Table S4). In the case of the latter, the three genes encoding RT-like domains are all paralogs of each other (KEGG Database) and similarly upregulated (>29–35-fold, Table S4). Further inspection of corresponding entries for these gene products in UniProt and InterPro revealed predicted retrotransposon gag, aspartic proteinase, RT, RNase H, and integrase domains, indicating they may indeed encode retrotransposon polyproteins. The domain organization and motifs are consistent with retrotransposons within the family *Belpaoviridae* [106] (also referred to as Bel/Pao, Class I retrotransposons based on previous classification systems [107]). It should be noted that the upregulation of retrotransposons and other transposable elements in response to infection has been observed in other organisms [108,109,110], and to the best of our knowledge this is the first description of this in a zebrafish model. Interestingly, upregulation of class I retrotransposons in zebrafish has also been observed in response to genome demethylation, leading to the induction of antiviral responses [111].

**Figure 10:**
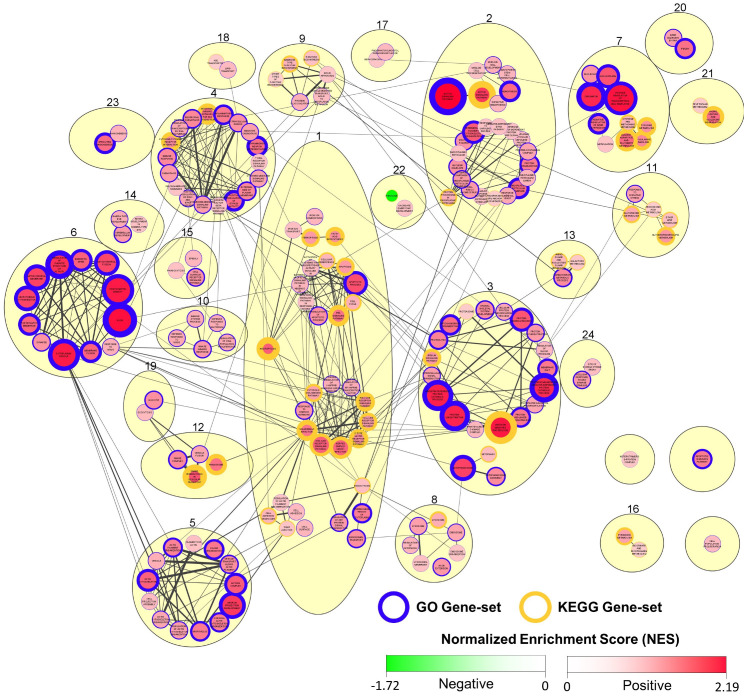
Summary of GSEA output indicating gene-set enrichment based on gene expression in CyHV-3-infected relative to mock-infected zebrafish larvae at 2 dpi. Cytoscape Network representing functional relationships between all significantly enriched gene-sets (positive or negative) identified in GSEA output (FDR adjusted *p*-value < 0.25). Nodes in the network represent GO (blue border) and KEGG Pathway (gold border) gene-sets. Edges (connecting lines) between nodes represent the similarity coefficient (measuring the functional/gene overlap between pairs of gene-sets). Edge thickness corresponds to magnitude of similarity coefficient (only edges with coefficient ≥2 are displayed). Each gene set exhibits either a positive or negative normalized enrichment score (NES), indicating predominant upregulation or downregulation of constituent genes, respectively. Accordingly, node colour and size both represent NES magnitude (exponentially transformed scale), with positive and negative enrichment represented by red and green, respectively, according to the colour scale provided. The node border thickness indicates the significance of enrichment (inverse of FDR adjusted *p*-values, thus the lower the FDR adjusted *p*-value, the greater the thickness). Using the MCL cluster algorithm, GO and KEGG gene-sets were clustered together based on their functional similarity as indicated by similarity coefficients (beige ovals), and numbers were assigned to each cluster. For the purposes of visual clarity, clusters were manually repositioned, and within some clusters, sub-clusters were manually grouped based on functional similarity. Clusters that are overlapping or touching in the absence of any visible edges between their respective nodes have shared edges below the 0.2 coefficient cut-off for display. Clusters that do not exhibit edges between their respective nodes and are also not touching or overlapping either have no common edges or have common edges with similarity coefficient >0.1. Higher quality figures for the whole manuscript are available in the PDF version.

In further analysis, we expanded our investigation to all genes included in differential expression analysis at 2 dpi (Table S5), exploring the response to infection at a “gene-set level”. Using GSEA, we identified GO and KEGG pathway gene-sets that were to a significant extent positively or negatively enriched in CyHV-3-infected larvae at 2 dpi (Tables S8 and S9, Figure S6). Cytoscape was used to generate a network of these significantly enriched gene-sets based on the functional relationships between them (Figure 10), providing a greater insight into what biological processes are implicated in the response to CyHV-3 infection in zebrafish larvae, and how they are related. Notably, only one gene-set, “Ribosome” (DRE03010), was found to be significantly negatively enriched, with all other significant gene-set responses involving positive enrichment. During the process of generating the network presented in Figure 10, nodes (i.e., gene-sets) were clustered together based on their similarity coefficient (related to gene-set/functional overlap). This process resulted in the formation of several large clusters, which we numbered. Cluster-1 is the largest of these and exhibits the highest quantity of functional connections with surrounding clusters, and as such, it represents a major aspect of the response to CyHV-3 infection. Within Cluster-1, there are two main sub-clusters. One of these is dominated by gene-sets related to programmed cell death, the other is dominated by PRR signalling, pathogen and inflammatory response gene-sets. Notably, enrichment of the RIG-I-like signalling pathway, the Toll-like receptor signalling pathway, and the Herpes simplex virus 1 gene-sets are consistent with zebrafish larvae response to NNV infection [78]. In Cluster-1, the KEGG Necroptosis pathway (DRE04217) is the most significant positively enriched gene-set, and joint most significantly enriched gene-set overall (Tables S8 and S9). Notably, this pathway gene-set is functionally related to other gene-sets in the apoptosis and PRR/inflammatory/pathogen response sub-clusters (manually isolated from these two sub-clusters in Cluster-1, Figure 10), exhibiting gene overlap with 15/19 of these gene-sets, with eight of these resulting in similarity coefficients >0.02 and thus displayed in Figure 10. This reflects the substantial crosstalk that exists between programmed cell death and PRR signalling in response to infection [67,112].

The prominence of positively enriched necroptosis and apoptosis related gene-sets in Cluster-1 supports the hypotheses derived from earlier observations in vitro and in vivo (Figure 6 and Figure 8 and Video S1), that apoptosis-like and non-apoptosis-like programmed cell death feature heavily in the zebrafish response to CyHV-3 infection. One of the important genes in the necroptosis pathway is *eif2ak2* (or *pkr*). It was identified as one of the main genes contributing to the enrichment signal for the necroptosis gene-set (Figure S7). It represents an important link between the innate immune response and the initiation of necroptosis [113]. This gene encodes a protein referred to as “interferon-induced, double-stranded RNA-activated protein kinase”, or more commonly, “Protein Kinase R” (referred to as PKR hereafter). PKR functions as both a general cellular stress sensor and PRR. Thus, it plays a diverse role in the innate immune response to viral infections and many fundamental cellular processes including programmed cell death [114].

**Figure 11:**
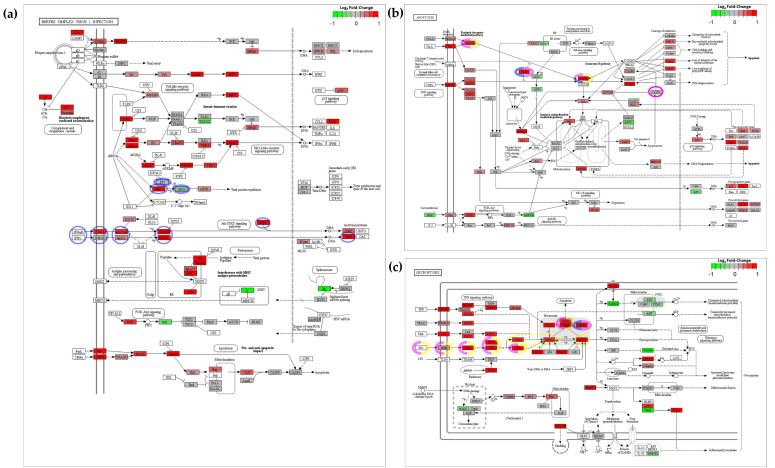
Visualization of differential gene expression in CyHV-3-infected zebrafish larvae (2 dpi) within KEGG pathway maps. Using the R package Pathview, gene expression data from our experiment was mapped to corresponding nodes in KEGG pathways (**a**) Herpes simplex virus 1 infection (**b**) Apoptosis and (**c**) Necroptosis pathways. Nodes represent zebrafish homologs of genes known to be involved in each pathway, with colour representing the log_2_-fold-change in gene expression in CyHV-3-infected relative to mock infected zebrafish larvae. Upregulated and downregulated genes are represented by red and green shades respectively, according to scale in the top right of each pathway. For visual clarity (due to large differences in fold change between genes) the maximum and minimum values in the colour scale is set at –1 and 1 log_2_-fold-change (corresponding to a two-fold change). It should be noted that many nodes represent combined differential expression from several zebrafish paralogs, thus the generic KEGG gene symbols are used as node names, which relate to the common names used to refer to protein products at each node. Not all the paralogs represented by each node are significantly differentially regulated. The list of zebrafish orthologs/paralogs corresponding to each node in these pathways can be accessed in the KEGG database using the corresponding gene-set references (Herpes simplex virus 1 infection (DRE05168), Apoptosis (DRE04210) and Necroptosis (DRE04217)), which can then be cross-referenced with data in Table S5 (using NCBI/Entrez/GenBank Gene IDs or Gene Symbols). Key genes involved in IFN-stimulated PKR-mediated programmed cell death, i.e., translational inhibition [114,116,128] leading to apoptosis [112] (blue), IFN-stimulated PKR-mediated apoptosis [129,130] (pink), and IFN-stimulated PKR-mediated necroptosis [113] (yellow) are highlighted. Genes with dashed line borders indicate instances where downregulation, translational inhibition or post-translational inactivation of protein products promote the processes in question (see main text and references provided within this caption for details). White nodes represent instances where zebrafish homologs have not been assigned thus far, or where gene expression from zebrafish homologs have not been detected. Higher quality figures for the whole manuscript are available in the PDF version.

PKR-mediated programmed cell death is important for the clearance of viral infections [113,115,116]; however, the antiviral roles of PKR are diverse. It also contributes to the antiviral actions of other enriched gene-sets within Cluster-1 (Figure 10). For example, in the “Herpes simplex virus 1” response gene-set (DRE05168), PKR is activated by dsRNA formed during infection, and subsequently phosphorylates eIF2α (its main substrate), resulting in the stalling of mRNA translation [114,115,117] (Figure 11a). However, some mRNA species are less affected by this [118,119,120]. This translational stalling also leads to the formation of stress granules (SGs) [121,122,123], which in some cases are important for detection of viral RNA via PRRs as in the “RIG-I-like receptor signalling pathway” (DRE04622) [124,125]. Furthermore, PKR also facilitates/promotes the NF-κB pathway, indirectly [114,118]. While this induces a pro-inflammatory response which may be useful in terms of counteracting infection, the accompanying pro-survival response (although helpful to some aspects of immune-response [126]), is counter to the pro-apoptotic function of PKR, but may act to only temporarily delay cell death [127]. Notably, expression from the zebrafish *nfkb1* gene, which encodes the zebrafish NF-κB ortholog, was not significantly upregulated at 2 dpi in our model (Table S5 and Figure 11b).

PKR-mediated apoptosis can occur via the “extrinsic” FADD-caspase-8 mediated pathway [131]. The circumstances under which this occurs are quite diverse. For example, PKR-mediated translational inhibition leads to apoptosis [115,116] via depletion of cFLIP protein [112] which acts as an important inhibitor of caspase-8 (Figure 11b) [132,133]. PKR phosphorylation by PACT (in response to stress) can also lead to translational inhibition leading to caspase-8 dependent apoptosis [134], as can overexpression of PKR [135,136,137]. In addition to IFN stimulation leading to upregulation of PKR, IFN-stimulated PKR-mediated apoptosis can also occur via JAK/TYK-mediated phosphorylation of PKR [129]. Notably, along with *eif2ak2* (encoding PKR), many other zebrafish genes encoding orthologs of ISGs involved in IFN-stimulated PKR-dependent apoptosis are also upregulated at 2 dpi in our model (Figure 11b,c). In parallel, PKR may also promote caspase-9 mediated apoptosis via the “intrinsic” apoptosis pathway. However, unlike caspase-8, caspase-9 was not upregulated at 2 dpi in our experiment (Figure 11b), indicating, as with other viral-host models [118,130,131], that caspase-8 mediated apoptosis also plays a more dominant role in response to infection in the CyHV-3-zebrafish larvae model.

Many viruses have evolved ways to interfere with apoptosis by disrupting elements of the FADD-caspase-8 pathway [72,114,138,139]. To counteract this, necroptosis may have evolved as a back-up mechanism of programmed cell death [72], which can occur via compromising of the cell membrane though action of MLKL [140] and/or production of reactive oxygen species [141]. This relies on the interaction of RIPK1 and RIPK3 for necrosome formation, a process that is inhibited by the FADD-caspase-8 complex [72,141,142]. Like apoptosis, PKR-mediated necroptosis can occur in response to IFNs, possibly requiring PKR interaction with RIPK1 [113]. While other groups have also observed a physical association between PKR and RIPK1 [143], the exact role that PKR plays in initiating necroptosis in response to IFN stimulation remains unclear [144]. Notably it has been proposed that IFN-stimulated PKR-mediated necroptosis is restricted to the G2M stage of the cell cycle, when FADD is disabled, preventing capase-8 inhibition of necrosome formation [113]. Given that in zebrafish larvae, and to lesser extent, in ZF4 monolayers, we expect widespread, frequent occurrence of mitosis, our models may be particularly predisposed to this type of PKR-mediated necroptosis. Notably, in addition to PKR itself, genes encoding zebrafish orthologs of ISGs involved in PKR-mediated necroptosis are also upregulated at 2 dpi (Figure 11c).

The *eif2ak2* gene encoding PKR was also among the top 250 most significant DEGs in this study (Table S4) and identified as an important hub gene in functional network in Figure 9, being ranked 3rd overall (Table S7). Given the importance of this ISG in terms of antiviral defence [112,115,145], particularly regarding programmed cell death, we hypothesized that the knock-out (KO) of the *eif2ak2* gene may impact CyHV-3 clearance in zebrafish larvae.

Unlike other vertebrates, members of the teleost fish families *Salmonidae* and *Cyprinidae* also encode an additional PKR-like protein referred to as “protein kinase containing Z-DNA binding domains” (or PKZ) [88,89,91]. PKZ genes may have evolved through duplication of the PKR encoding genes in these teleost fish families, after divergence from tetrapods [88]. Consequently, PKZ exhibits a high degree of sequence similarity to PKR proteins encoded in the same genomes, predominantly to the C-terminal kinase domain, which is responsible for eIF2α phosphorylation by PKR [89,146].

However, unlike PKR, PKZ contains Zalpha (Zα) domains instead of dsRNA binding domains in the N-terminal [146] (Figure 1). These domains are capable of binding to Z-DNA/RNA, which exist in the left-handed double helix conformation as opposed to the more common right-handed conformation of dsDNA/RNA (referred to as A and B-DNA/RNA) [90].These two features indicate that: (1) Like PKR, PKZ acts as an eIF2α kinase and mediates translational stalling, and induction of apoptosis via eIF2α phosphorylation [88,89,147,148], and (2) Like PKR, PKZ acts as a cytosolic PRR, but is activated by a greater diversity of nucleic acids than PKR. PKZ nucleic acid binding, B-to-Z conversion, and PKZ-mediated translational stalling have been best demonstrated using B and Z-DNA [89,149,150,151,152], indicating co-operative antiviral roles for PKZ and PKR. However, given that the Zα domains of PKZ do bind to RNA analogues [153] and that some Zα domains exhibit A-to-Z RNA conversion (as we recently demonstrated [90]), like PKR, PKZ may also detect and be activated by dsRNA. Thus, PKZ may provide at least some degree of back-up for PKR, leading to some redundancy among zebrafish IFN induced eIF2α kinases.

Notably, the *pkz* gene (encoding PKZ) was the 23rd most significantly upregulated gene at 2 dpi in our model, upregulated more than the *eif2ak2* gene (encoding PKR, ranked 250th, Table S4), and the *pkz* expression levels were >3 fold higher. In addition, *pkz* was ranked 9th in hub gene analysis in Figure 9 (see also Table S7). Given their potentially overlapping functions, in addition to the PKR-KO mutant (lacking *eif2ak2*), we generated a separate mutant, PKZ-KO (lacking *pkz*, Figure 1), to investigate the importance of both these multifunctional eIF2α kinases in the clearance of CyHV-3 in zebrafish larvae.

### 3.5. The Absence of PKR and/or PKZ Does Not Impair the Clearance of CyHV-3 Infections in Zebrafish Larvae

**Figure 12:**
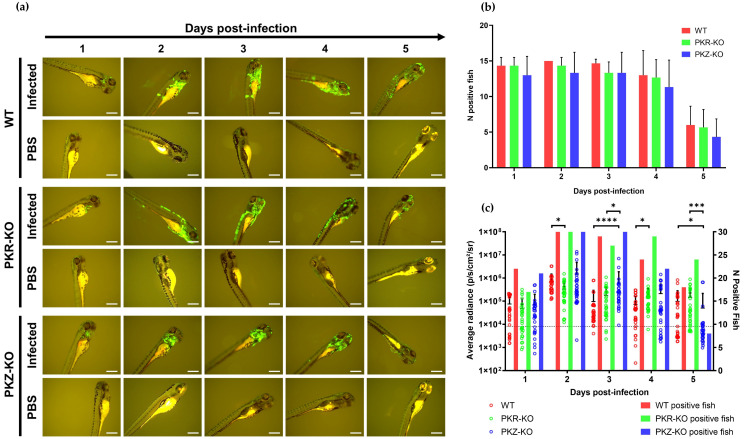
Replication of CyHV-3 in different zebrafish strains. (**a**) Epifluorescence microscopy images representative of larvae inoculated by microinjection with either CyHV-3 EGFP or mock-inoculated with PBS according to time postinfection (longitudinal observation of the same larvae over all timepoints). For all strains infection clearance commenced from 4–5 dpi. Scale bars = 500 µm. (**b**) Numbers of infected larvae among zebrafish strains inoculated with CyHV-3 EGFP (n = 15). Data represents mean ± standard errors from 3 independent experiments (longitudinal observation of the same larvae over all timepoints). (**c**) IVIS analysis measuring Luc2 expression in different zebrafish strains microinjected with CyHV-3 Luc (n = 30). The data points represent the mean radiance per larvae according to time postinfection with mean ± standard error represented for each group at each timepoint. The discontinuous line represents the cut-off for positivity and the mean + 3 × SD of the values obtained for mock-infected larvae. The number of positive larvae at each timepoint is represented by bars. * *p* < 0.05; *** *p* < 0.001; **** *p* < 0.0001.

In vitro and in vivo experiments performed in this study indicated that CyHV-3 infection was rapidly cleared in zebrafish models via programmed cell death. This was supported by the transcriptomic analysis from infected larvae, which also supported a potentially important role for the eIF2α kinases PKR and PKZ in this process. Based on this evidence, we tested the impact of these eIF2α kinases on CyHV-3 clearance using CRISPR/Cas9 generated PKR-KO and PKZ-KO zebrafish mutants (Figure 1). Mutant and WT zebrafish larvae were first infected with CyHV-3 EGFP by microinjection as per earlier experiments. As we hypothesized that the onset of infection clearance may take longer to occur in eIF2α kinase KO mutants, we also extended the monitoring period from 4 dpi (in earlier experiments) to 5 dpi. Epifluorescence microscopy suggested that PKR-KO and PKZ-KO mutants cleared viral infection as efficiently as WT larvae (Figure 12a). There was also no difference between the zebrafish strains in terms of the numbers of infected larvae at each timepoint (Two-way RM ANOVA, *p*-value = 0.6440), with all groups exhibiting a dramatic decrease in the number of positive fish at 5 dpi (Figure 12b).

Next, WT, PKR-KO, and PKZ-KO zebrafish strains were infected with CyHV-3 Luc as before, allowing viral replication to be compared between strains (Figure 12c). This revealed no significant difference in viral signal between the three zebrafish strains (Durbin Test, *p*-value = 0.6500). Relative differences in signals between the WT and PKR-KO strains were inconsistent over the monitoring period, with no clear trends to indicate a difference between the two strains. In contrast, virus levels in the PKZ-KO strain were consistently higher than both WT and PKR-KO strains from 1–4 dpi, with significant differences at 3 dpi. However, viral levels in PKZ-KO larvae were significantly lower than other strains by 5 dpi (Figure 12c), indicating greater clearance, despite higher viral levels from 1–4 dpi.

Cognisant of the possible redundancy in eIF2α kinase function (described earlier), which may have allowed PKZ to compensate for the absence of PKR in the PKR-KO mutant, and vice versa, we generated a third mutant, PKR-PKZ-KO, lacking both *pkz* and *eif2ak2* genes (Figure 1). This strain was included in an additional experiment, like the one presented in Figure 12. (Figure S8). However, surprisingly, the viral loads observed in the PKR-PKZ-KO mutants were not significantly different from the WT strain. Taken together, the results from these two experiments indicate that 1) PKR and PKZ are not essential for clearance of CyHV-3 infection in zebrafish larvae, and 2) even at this early developmental stage, the zebrafish immune system exhibits sufficient redundancy to enable clearance of CyHV-3 infection in the absence of PKZ and/or PKR.

If programmed cell death also features heavily in the response to CyHV-3 infection in these mutant zebrafish strains, as earlier observations in the WT strain suggested (Figure 8, Figure 11 and Video S1), these processes would need to be mediated via other mechanisms. Notably, in addition to IFN-stimulated PKR/PKZ-mediated programmed cell death [112,113,129], these processes can be stimulated by other cytokines such as FAS, TNFα, and TRAIL [154,155,156,157] (the zebrafish orthologs for these proteins are encoded by the *faslg*, *tnfa*, and *tnfsf10* genes, respectively). Like IFN, these cytokines also operate by binding to their respective cell membrane receptors and downstream interactions between these and various other proteins are required to initiate apoptosis and/or necroptosis. Notably, genes encoding zebrafish orthologs of most of the proteins involved in these processes are also upregulated in response to infection at 2 dpi (Figure 11), indicating some potential redundancy in terms of the programmed cell death response. However, no expression from the *faslg* and *tnfa* genes was observed in our model. While we did observe expression for *tnfsf10*, it was not upregulated in response to infection. Therefore, similar to what we have hypothesized regarding IFN expression kinetics, it is possible that with this model, the upregulation of these three cytokines is also extremely brief, occurring very early after infection with a rapid return to basal levels after. As with IFN, further investigation will be needed to establish the expression kinetics of these cytokines in response to CyHV-3 infection in this host model, and to what extent, if any, they contribute to programmed cell death and clearance of CyHV-3 infection.

In both experiments (Figure 12 and Figure S8), the PKZ-KO mutant exhibited a higher viral load than other strains at the earlier stages of infection. The higher levels of CyHV-3 in the absence of PKZ may indicate the importance of host Zα domain-containing PRRs such as PKZ, in restricting CyHV-3 in the early stages of infection. This is consistent with our recent study where we provide strong evidence that the CyHV-3 ORF112 protein, which also contains a Zα domain, acts as an essential antagonist of RNA PRRs during CyHV-3 infection [90]. However, the absence of PKZ still leads to more dramatic viral clearance at 5 dpi relative to PKZ-competent strains (Figure 12c). We hypothesize that higher viral replication, from 1–4 dpi, may have ultimately led to an increased innate immune response, priming a more dramatic clearance at 5 dpi. Even if the absence of PKZ does not prevent viral clearance, the higher levels of viral replication in earlier stages, may lead to increased tissue damage via potential inflammatory response, which may ultimately be harmful to the host. Therefore, having the complete repertoire of PRRs necessary for effective restriction of CyHV-3 replication prior to clearance may still be important. Surprisingly, we do not observe higher viral loads at earlier stages of infection in the PKR-PKZ-KO mutant (also lacking PKZ), which instead exhibited a similar phenotype to WT and PKR-KO strains in response to CyHV-3 (Figure S8). These observations open up several interesting avenues for further investigation, in particular the characterization of the innate immune response in zebrafish mutants lacking these important PRRs and the possible impact of reduced eIF2α phosphorylation on programmed cell death, if any, in response to CyHV-3 infection in this model.

## 4. Conclusions

The aim of this present study was to investigate the potential of the zebrafish model to study AngHV-1, CyHV-2, and CyHV-3, which are three economically important viruses in the family *Alloherpesviridae*. We conclude that while the zebrafish ZF4 cell line is moderately susceptible to these three viruses, it is less susceptible and not permissive to AngHV-1 (Figure 2). ZF4 cells do exhibit transient permissiveness to CyHV-2 and CyHV-3 infection. These cells are more permissive to CyHV-3, but both viruses exhibit inefficient cell to cell viral transmission in this in vitro model (Figure 3 and Figure 4). These viruses are ultimately cleared from ZF4 monolayers, in a process which is preceded by what resembles widespread programmed cell death among infected cell populations (Figure 3, Figure 4 and Figure 6). As zebrafish larvae were not susceptible to these viruses via inoculation by immersion, we conclude that these viruses may not be capable of entering zebrafish larvae through natural routes in vivo (Figure 7). However, zebrafish larvae are susceptible to infections with CyHV-2 and CyHV-3 via microinjection, an artificial inoculation route (Figure 7). Conversely, we conclude that zebrafish larvae are not susceptible to AngHV-1 via both inoculation methods used in this study (Figure 7). This lower susceptibility to AngHV-1 in vitro and in vivo, may reflect the fact that, unlike CyHV-2 and CyHV-3, AngHV-1 does not naturally infect host species from the family *Cyprinidae*. Even though larvae exhibit greater susceptibility to CyHV-2 and CyHV-3, we conclude that these infections are rapidly cleared (Figure 7 and Figure 12). We also conclude that zebrafish larvae exhibit more susceptibility (and possibly more permissivity) to CyHV-3, given higher viral levels and slower clearance, indicating the superior utility of this virus-host model in future studies. Interestingly, given that strains within each cyprinivirus species clad exhibit natural heterogeneity regarding replication in vitro and/or in vivo (at least with AngHV-1 and CyHV-3 [40,74]), it remains possible that the use of alternative cyprinivirus strains with the same zebrafish models may result in different outcomes, and is something which remains to be explored in the future.

As we observed transcription of all 155 known CyHV-3 protein coding genes in infected zebrafish larvae (Figure S3, Table S3), we conclude that zebrafish cells may be permissive to CyHV-3 replication in vivo. However, unlike infections in vitro, we observed no clear evidence of CyHV-3 transmission to new cells prior to clearance in vivo (Figure 8, Video S1). Thus, the extent to which this permissiveness leads to successful CyHV-3 transmission between cells in vivo remains unclear.

As per observations in vitro, CyHV-3 clearance in zebrafish larvae is also preceded by apoptosis-like death among infected cells (Figure 8, Video S1). These infections stimulate the upregulation of many ISGs (Figure 9, Tables S4 and S5). The upregulation of genes involved in programmed cell death and nucleic acid sensing PRR pathways represent a core part of this response (Figure 10 and Figure 11). PKR and PKZ are also upregulated in response to infection (Figure 9, Table S4) and may contribute to both programmed cell death and nucleic acid sensing PRR pathways (Figure 10 and Figure 11). However, their absence in mutant zebrafish strains does not impact CyHV-3 clearance (Figure 12). This may be due to sufficient levels of redundancy within the zebrafish innate immune response processes, even at this early developmental stage (Figure 11). Interestingly, CyHV-3 may represent an ideal model to utilize in the study of viral clearance by the innate immune system in this important and widely studied host. This opens many interesting avenues for future investigation to determine what elements of the immune response are essential for this process. As part of this, the generation of new KO mutants, guided by the transcriptomic data generated in this study, may lead to the development of zebrafish strains that are more permissive to these economically important viruses, which may themselves be utilized as valuable research tools in the future.

This is the first report of the generation and use of PKR and/or PKZ KO zebrafish mutants (Figure 1 and Figure 12), and they will represent useful subjects for further characterization and the study of other viruses in zebrafish models. Given the importance of PKR, and potentially PKZ, in the innate immune responses and in many more cellular processes, and the widespread use of zebrafish as a model organism, the KO mutants generated in this study will be of interest to many more researchers in the wider field. Thus, sperm corresponding to these mutants will be deposited in the European Zebrafish Resource Centre (EZRC) for ease of distribution elsewhere.

Furthermore, we note that many of the most significantly upregulated genes in response to CyHV-3 infection in zebrafish larvae were uncharacterized, and some were previously unreported as being involved in the immune response (Table S4). These include five non-coding transcripts (one of which was >3000-fold upregulated and the 6th most upregulated gene at 2 dpi). We propose to provisionally refer to these five transcripts as “Zebrafish Non-coding Infection Response Element” 1–5 (or ZNIRE 1–5, complete details in Table S4). This observation was particularly intriguing, and we propose that further research into their importance during the immune response will be necessary. We also observed the upregulation of three retrotransposons (all ~30-fold upregulated, Table S4). It is possible that this retrotransposon re-activation/upregulation in response to infection may be beneficial. Their cytoplasmic RNA and/or DNA genome intermediates may potentially act as ligands for PRRs [111], thus enhancing the innate immune response to viral infection and presenting an interesting hypothesis for further study with our model.

## Data Availability

All RNA-Seq data generated in this study is publicly available on https://www.ncbi.nlm.nih.gov/under BioProject Accession number PRJNA929940 (accessed on 13 March 2023).

## Supplementary Materials

The following supporting information can be downloaded at: https://www.mdpi.com/article/10.3390/v15030768/s1, Figure S1. Production and characterization of the CyHV-3 EGFP recombinant strain as described in Methods S1; Figure S2. Evolution of fluorescence intensity in infected ZF4 cells; Figure S3. Box Plots of read counts mapping to each known CyHV-3 open reading frame (ORF) at 2 dpi; Figure S4. Volcano plots of summarizing DEGs at each timepoint; Figure S5. CytoHubba Analysis of STRING protein query network; Figure S6. Summary of the top 40 positively enriched GO gene-sets; Figure S7. Leading edge analysis of the necroptosis pathway; Figure S8. Comparison of CyHV-3 replication and clearance between PKR-PKZ-KO and other zebrafish strains; Table S1. Primers used in this study; Table S2. Summary of DEGs from each timepoint; Table S3. Read Counts mapping to each known CyHV-3 Open Reading frame (ORF) at 2 dpi; Table S4. Top 250 significant DEGs in zebrafish larvae infected with CyHV-3 at 2 dpi; Table S5. Raw output of DESeq2 in zebrafish larvae infected with CyHV-3 at 2 dpi; Table S6. Summary of STRING enrichment analysis; Table S7. STRING CytoHubba Ranking; Table S8. Summary of Gene Ontology (GO) enrichment analysis; Table S9. Summary of Kyoto Encyclopaedia of Genes and Genomes (KEGG) pathway gene set enrichment analysis; Video S1; Methods S1. Production of CyHV-3 ORF136 EGFP recombinant strain [158,159,160,161,162,163,164], and Methods S2. Bioinformatic analysis [165,166,167,168,169,170,171,172,173,174,175,176,177,178,179,180,181,182,183,184,185].
