# Supplementary material for: Susceptibility and Permissivity of Zebrafish (Danio rerio) Larvae to Cypriniviruses
Source: Viruses. 2023 Mar 17;15(3):768. doi: 10.3390/v15030768 (PMC10051318; doi:10.3390/v15030768)
Supplement: Supplementary file 1 [file viruses-15-00768-s001.zip › Streiff et al 2023 Post Proof Supplementary Methods.pdf]

Methods S1. Production of CyHV−3 ORF136 EGFP recombinant strain

In addition to the three premade strains outlined in the main article, a fourth strain, CyHV−3 FL BAC revertant ORF136 EGFP strain (referred to as CyHV−3 EGFP in the present manuscript) was constructed specifically as part of this study. In all experiments, CCB cells and CyHV−3 were both cultured at 25°C as described previously [50,158]

S1.1 Production of a ORF136−ORF137−EGFP recombinant plasmid

A modified pcDNA3−LUC vector, kindly provided by M. Bremont (INRA, France), previously used by our lab [50], was subject XbaI digestion, resulting in the excision of a luciferase ORF. The luciferase was replaced with and EGFP ORF. This was done by PCR amplification of an EGFP ORF using Phusion High−Fidelity PCR Master Mix (NEB) from a p3E−EGFPpA plasmid (tol2kit plasmid), kindly provided by Dr Isabelle Manfroid (GIGA Stem Cells − Zebrafish Development and Disease Model, University of Liège, Belgium). The primers used for this (Fw−EGFP−linkDH59 and Rev− Fw−EGFP−linkDH59) bore homology to regions (15 bp length) adjacent to their respective XbaI cut−sites in the pcDNA3−LUC vector facilitating insertion using NEBuilder, Hifi DNA Assembly Master Mix (NEB). This resulted in pcDNA3−EGFP vector containing an EGFP gene expression cassette, flanked by a Human cytomegalovirus (HCMV) promoter and poly 3'UTR/Polyadenylation signal. The pGEMT−136NheI137 vector, containing an insertion partially encompassing CyHV−3 ORF135, ORF136 and ORF137 (nucleotides 234511−235330 of the CyHV−3 Luc genome, GenBank accession no. KP343683.1) with an NheI restriction site inserted into the intergenic region between CyHV−3 ORF 136 and ORF 137 (previously used by our lab [50]), was Digested by NheI. The EGFP expression cassette was amplified by PCR from the in pCDNA−EGFP vector as described previously. The primers used for this (Fw−CMV−EGFP−link PGEMT136/137 and Fw−CMV−EGFP− link PGEMT136/137) bore homology to regions (15 bp length) adjacent to the NheI cut site (with forward and reverse primers corresponding to nucleotides 231,413−231,422 and 231,423−231,432 of the CyHV−3 FL Luc genome respectively). This homology was used to guide insertion of the amplified EGFP expression cassette into the NheI−digested pGEMT−136NheI137 vector. This resulted in the creation of the pGEMT−136NheI137−EGFP vector expressing the EGFP reporter gene, in which the EGFP cassette is flanked by CyHV−3 sequences (Fig. S1a). All Primer sequences are provided in Table S1.

S1.2 Generation of CyHV−3 EGFP by homologous recombination between CyHV−3 Luc and pGEMT ORF136−ORF137−EGFP vector.

Freshly cultured CCB cells were transfected with the newly engineered pGEMT ORF136−ORF137−EGFP using Lipofectamine Plus as per manufacturers recommendation (Invitrogen) and incubated at 25°C for 24 hours (h). The same cell monolayers were then infected with parental CyHV3 Luc (GenBank accession no. KP343683.1) at the multiplicity of infection (MOI) of 1 plaque forming unit (pfu)/cell. Following an additional 5 days of incubation at the same temperature, single EGFP plaques were picked and sub−cultured. This was repeated three times until complete EGFP fluorescence was observed in the absence of luciferase signal over the entire cell monolayer.

S1.3 Genetic characterization of the CyHV−3 EGFP recombinant strain

The sequence of the CyHV−3 EGFP recombinant strain was confirmed by monitoring SacI restriction fragment length polymorphism (RFLP) by agarose gel electrophoresis and full−length genome sequencing as described previously [49] (Fig. S1b).

S1.4. Viral growth assay

Triplicate cultures of CCB cells were infected with each CyHV−3 strain (CyHV−3 WT, CyHV−3 Luc and CyHV−3 EGFP) at a MOI of 0.05 pfu/cell. After an incubation period of

2 h, the cells were washed with PBS and overlaid with culture medium. The infected cells were scraped off and collected with the supernatant at successive intervals (0, 2, 4 and 6 days postinfection (dpi)) and stored at −80°C. Titers of infectious viral particles were determined by triplicate plaque assays in CCB cells as described previously [50,158] (Fig. S1c).

S1.5. Viral plaque area assay

Triplicate cultures of CCB cells grown in 6−well plates were inoculated with three different isolates (CyHV−3 WT, CyHV−3 Luc and CyHV−3 EGFP) at an MOI of 200 pfu/well. After incubating for 2 h, the cells were washed with PBS and overlaid with culture medium supplemented with 1.2% (w/v) carboxymethylcellulose (CMC; medium viscosity, Sigma), in order to obtain isolated plaques [159]. At various times postinfection (pi), individual plaques were visualized by indirect immunofluorescence staining and imaged using a confocal microscope (Nikon A1R or Leica SP5), and areas were measured using ImageJ software [160].

Cells were fixed in phosphate buffered saline (PBS) containing 4% (w/v) paraformaldehyde (PAF) at 4°C for 15 min and then at 20°C for 10 min. After washing with PBS, samples were permeabilized in PBS containing 0.1% (v/v) Nonidet P−40 at 37°C for 15 min. Immunofluorescent staining (incubation and washes) was performed in PBS containing 10% (v/v) foetal calf serum (FCS). The mouse monoclonal antibody 8G12 raised against an unidentified CyHV−3 nuclear protein (diluted 1:500, v/v) was used as a primary antibody and was incubated at 37°C for 1 h. After washing with 10% v/v PBS−FCS, Alexa Fluor 488 goat anti−mouse (GAM) IgG1 (Invitrogen) and Alexa Fluor 546 goat anti−mouse (GAM) IgG2a (Invitrogen) were used as secondary antibodies (diluted 1:600) in 10% (v/v) PBS−FCS. The secondary antibodies were incubated at 37 °C for 30 min. After washing, the cells were mounted using Prolong Gold antifade reagent (Invitrogen) and analyzed by confocal microscopy (Fig. S1d).

Methods S2. Bioinformatic analysis

Before alignment, raw reads (in Fastq format) were processed using BBduk (v38.26) [161] facilitating adaptor sequence removal and quality trimming. This was followed by assessment of processed Fastq files using FastQC (v0.11.8) [162]. Processed reads were then aligned to the zebrafish reference genome GRCz11 (Ref Seq: GCF_000002035.6) using HISAT2 (v2.1.0) [163,164]. The resulting SAM files were converted to BAM format using SAMTools (v1.9) [165]. This mapping data was used as input for StringTie (v1.3.5) [163] which was used to conduct reference−guided transcript expression estimation.

Differential gene expression analysis between infected and mock samples at each timepoint was then conducted using DESseq2 (v1.36.0) [166]. Taking each time point (1, 2 and 4 dpi) separately, first, the python script PrepDE.py [167] was used to derive gene expression data from the StringTie output files and parse it into a format compatible with DESeq2. In the resulting CSV file, using a combination of Excel and awk (Linux) processing, the default StringTie gene identifiers were converted to Entrez Gene IDs using information from the corresponding genome GFF file. For non−unique Entrez Gene IDs (for example where the same genes on defined chromosomes also exist in alternate loci groups, or unplaced scaffolds), only data relating to the chromosome loci were retained. In instances where Entrez Gene IDs were non−unique but were only annotated on unplaced scaffolds, genes were eliminated from further analysis. After import of CSV files into R (v4.2.0) [62], in order to minimize false positives within differential expression output (caused by genes with extremely low or inconsistent expression i.e. absent in some replicates and detected at low levels in others), genes with read counts ≥10 in ≥3 samples were eliminated from DESeq2 analysis. Global, differential gene expression data from

DESeq2 output (infected relative to mock) was visualized using volcano plots, generated using EnhancedVolcanoPlot (v1.4.0) [168].

In order to ascertain the functions and biological processes associated with the most significantly differentially expressed genes (DEGs), the top 250 ranked DEGs (based on FDR adjusted *p*–value) were further analysed in Cytoscape (v3.8.0) [169]. This was done using the STRING Cytoccape application (v1.6.0) [170] which identifies known functional associations between genes, which is subsequently used generate protein interaction/functional association networks, facilitating the identification of the main biological responses to infection. This was also used to infer putative novel associations via node clustering patterns. Network layout was generated using GeneMania Force Directed Layout available as part of the GeneMania Cytoscape application (v3.5.2) [171]. Cytoscape was also used to analyse each of these networks using a Maximal Clique Centrality algorithm via the CytoHubba Cytoscape application (v0.1) [172], inferring the relative importance of each node within the functional association network.

Using the entire gene expression dataset, GSEA (v4.1.0) [173,174] was then used to identify enriched gene-sets. As expression data input for GSEA, normalized DESeq2 expression data was exported to tab delimited format, parsed to Gene Cluster Text file format (*.gct) as per Broad Institute definitions [175] and imported in the GSEA. As gene–set input for GSEA, lists of *Danio rerio* Gene Ontology (GO) [176,177] and Kyoto Encyclopedia of Genes and Genomes (KEGG) pathway [178–180] gene–sets (based on Entrez/NCBI/GenBank Gene IDs) were both retrieved and converted to Gene Matrix Transposed file format (*.gmt) using EnrichmentBrowser (v2.20.7) [181]. After running GSEA on expression data, EnrichmentMap (v3.3.1) [182] was used to generate a network of significantly enriched/depleted GO and KEGG pathway gene–sets (FDR adjusted *p*–value <0.25), based on similarity coefficients between gene–sets. These networks were viewed and analysed in Cytoscape. Gene–sets were grouped into clusters, with the MCL cluster algorithm implemented via the Cytoscape app AutoAnnotate (v1.3.3) [183]. KEGG pathways of interest were analysed further by mapping DEG data to pathway maps. This was done with Pathview (v1.36.0) [184] using Log_2–Fold change data from DESeq2 as input. Gene symbols and descriptions were retrieved using myGene (v1.34.0) [185]. Zebrafish orthologs of proteins of interest in this study were identified using the KEGG database. Statistical analysis associated with all bioinformatics tools that were used in this study are described in the respective publications associated with each tool.

Read counts for each CyHV−3 ORF were also generated using HISAT2, SAMTools, StringTie and PrepDE as described above, except reads were mapped to the CyHV−3 RefSeq genome (GenBank Accession Number: NC_009127.1)

Please refer to main manuscript for bibliography.
