# Supplementary figures and images for: Susceptibility and Permissivity of Zebrafish (Danio rerio) Larvae to Cypriniviruses
Source: Viruses. 2023 Mar 17;15(3):768. doi: 10.3390/v15030768 (PMC10051318; doi:10.3390/v15030768)

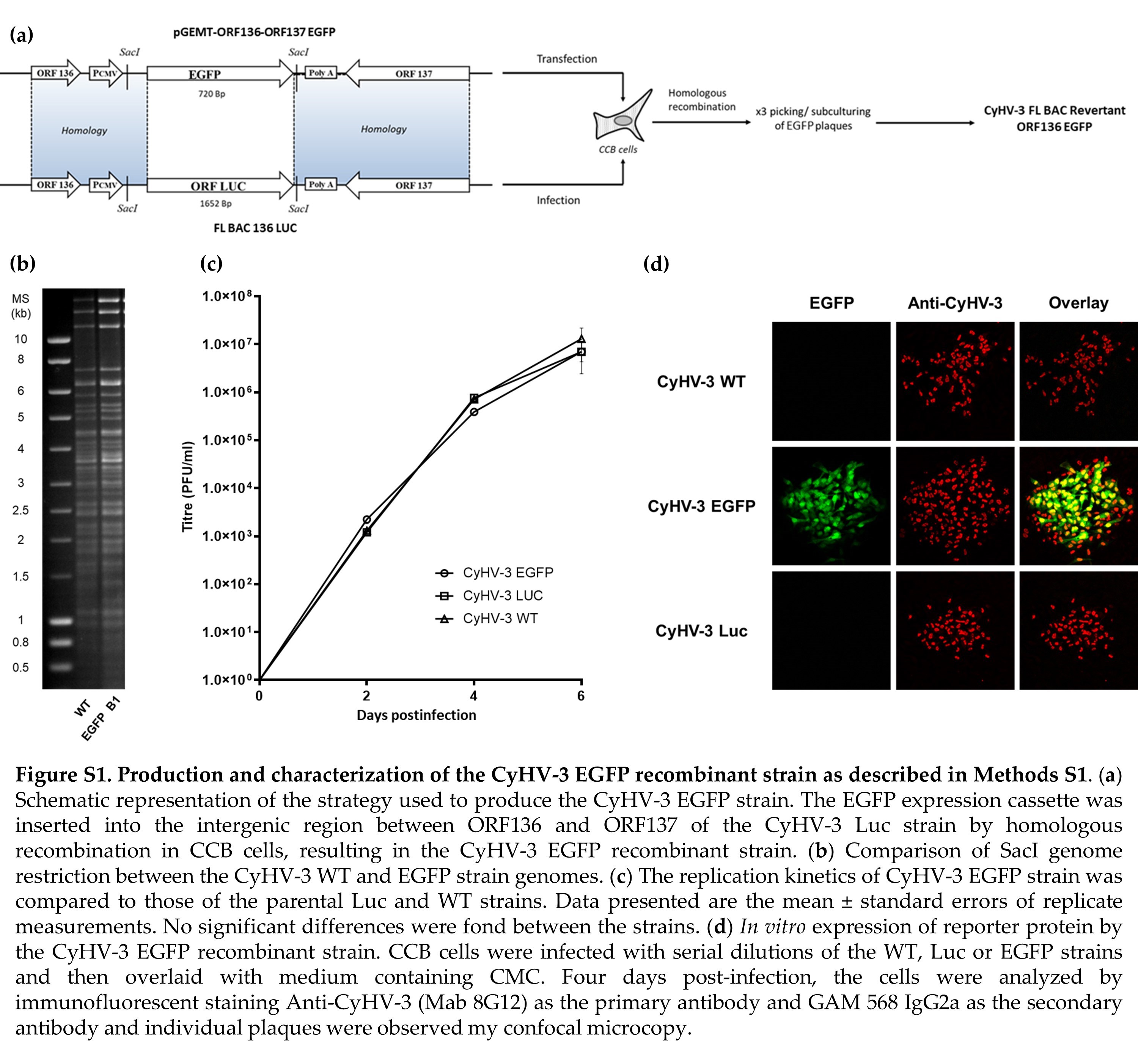

Supplement: Supplementary file 1 [file viruses-15-00768-s001.zip › Figure S1.JPG]

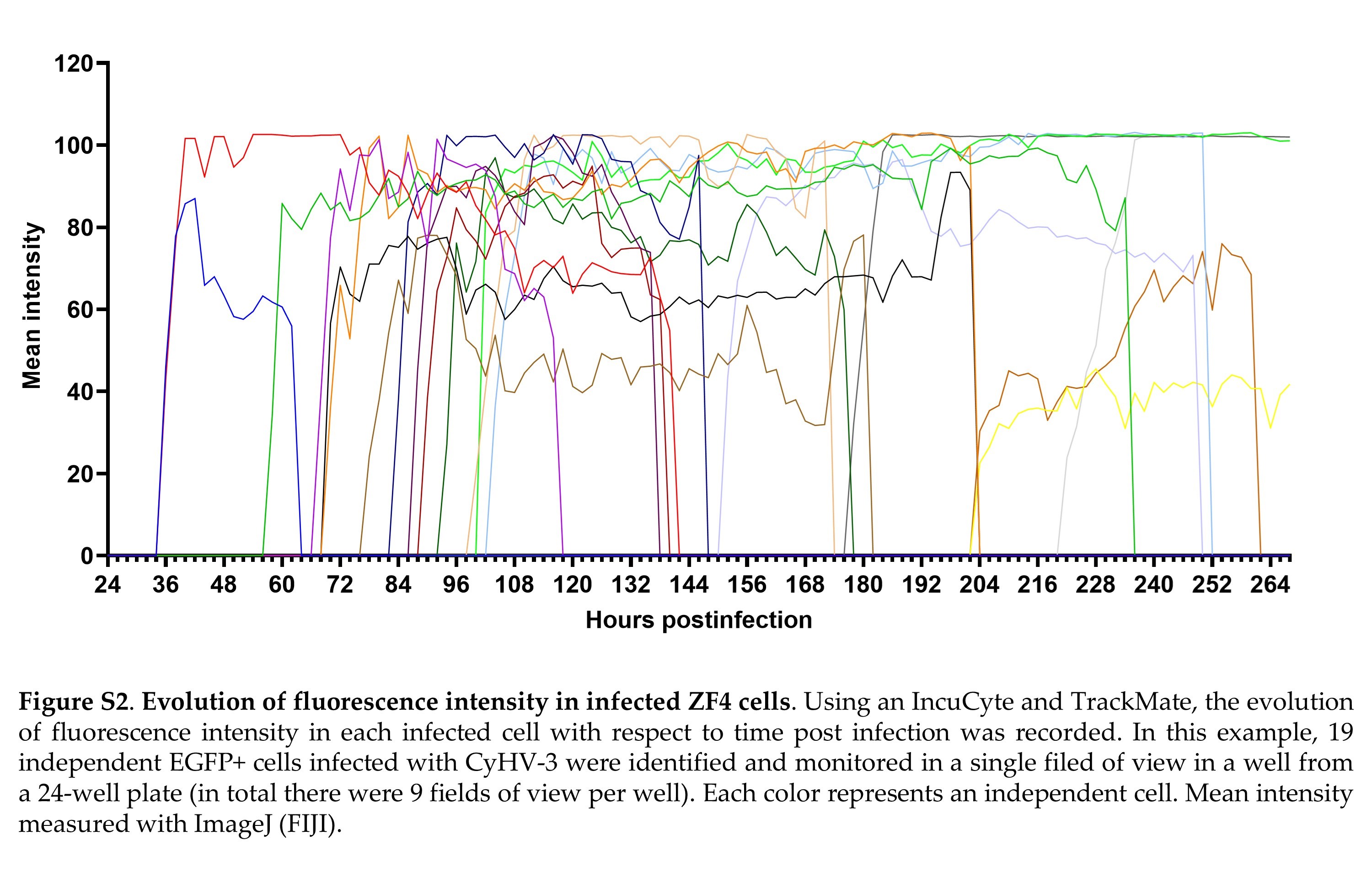

Supplement: Supplementary file 1 [file viruses-15-00768-s001.zip › Figure S2.JPG]

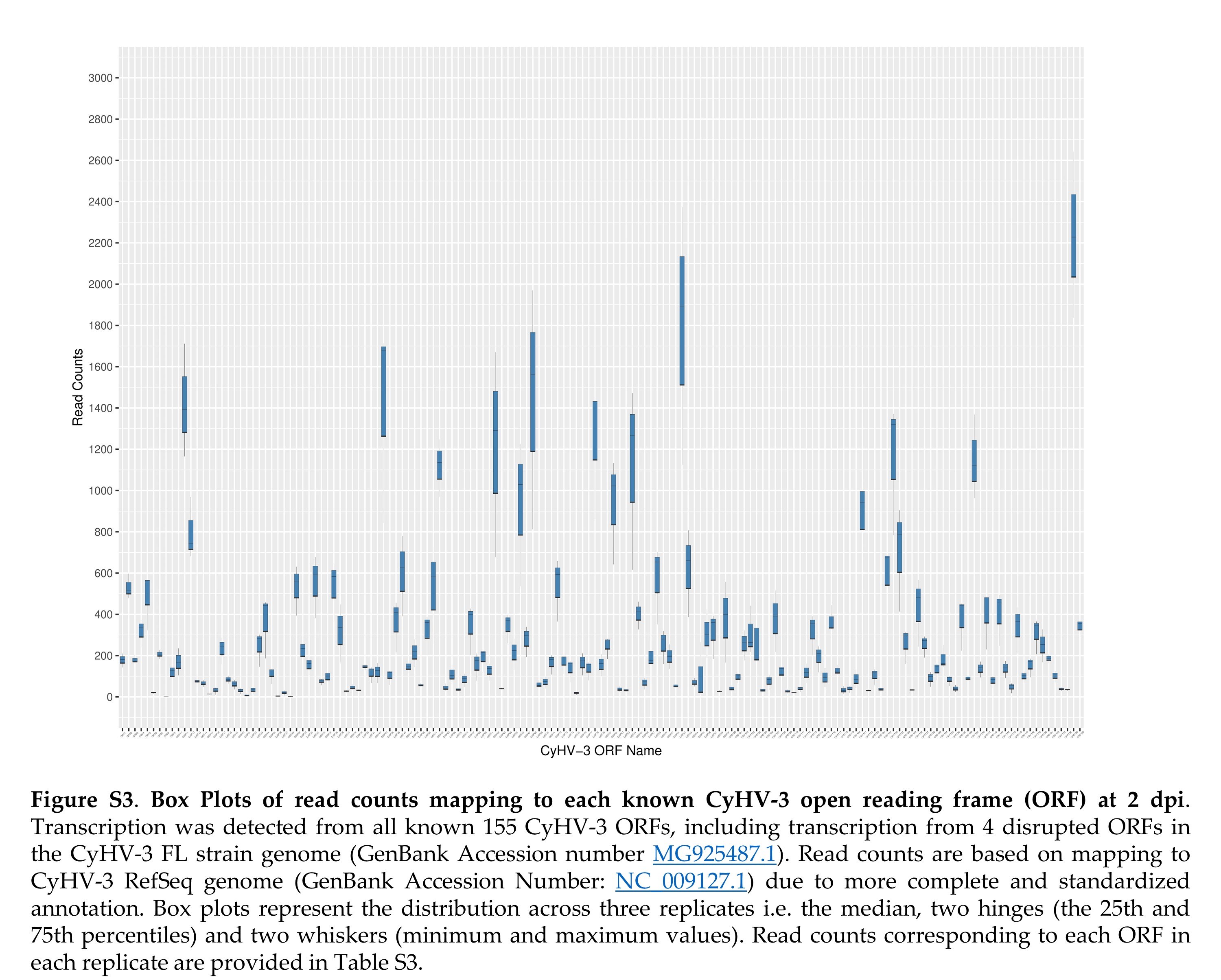

Supplement: Supplementary file 1 [file viruses-15-00768-s001.zip › Figure S3.JPG]

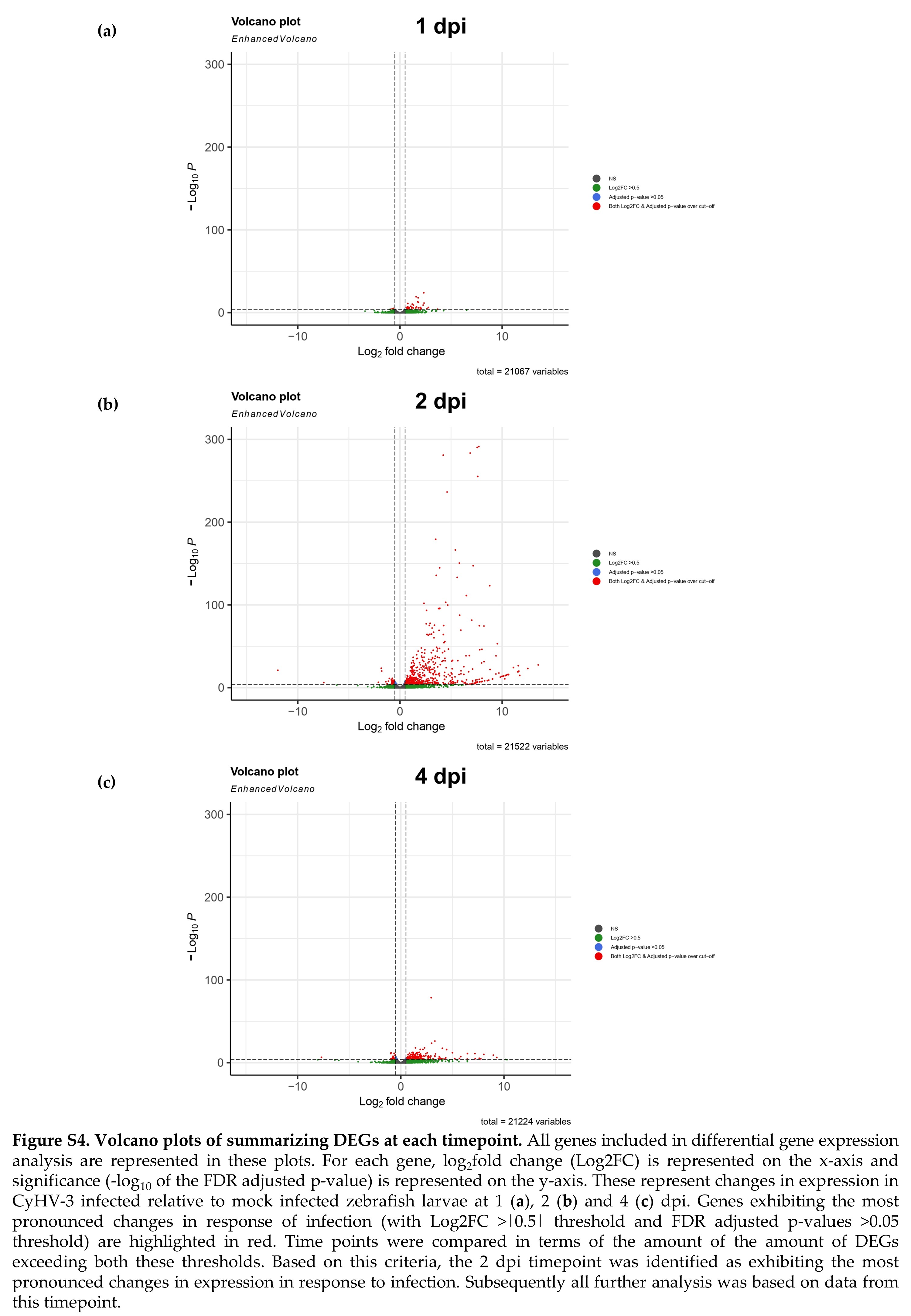

Supplement: Supplementary file 1 [file viruses-15-00768-s001.zip › Figure S4.JPG]

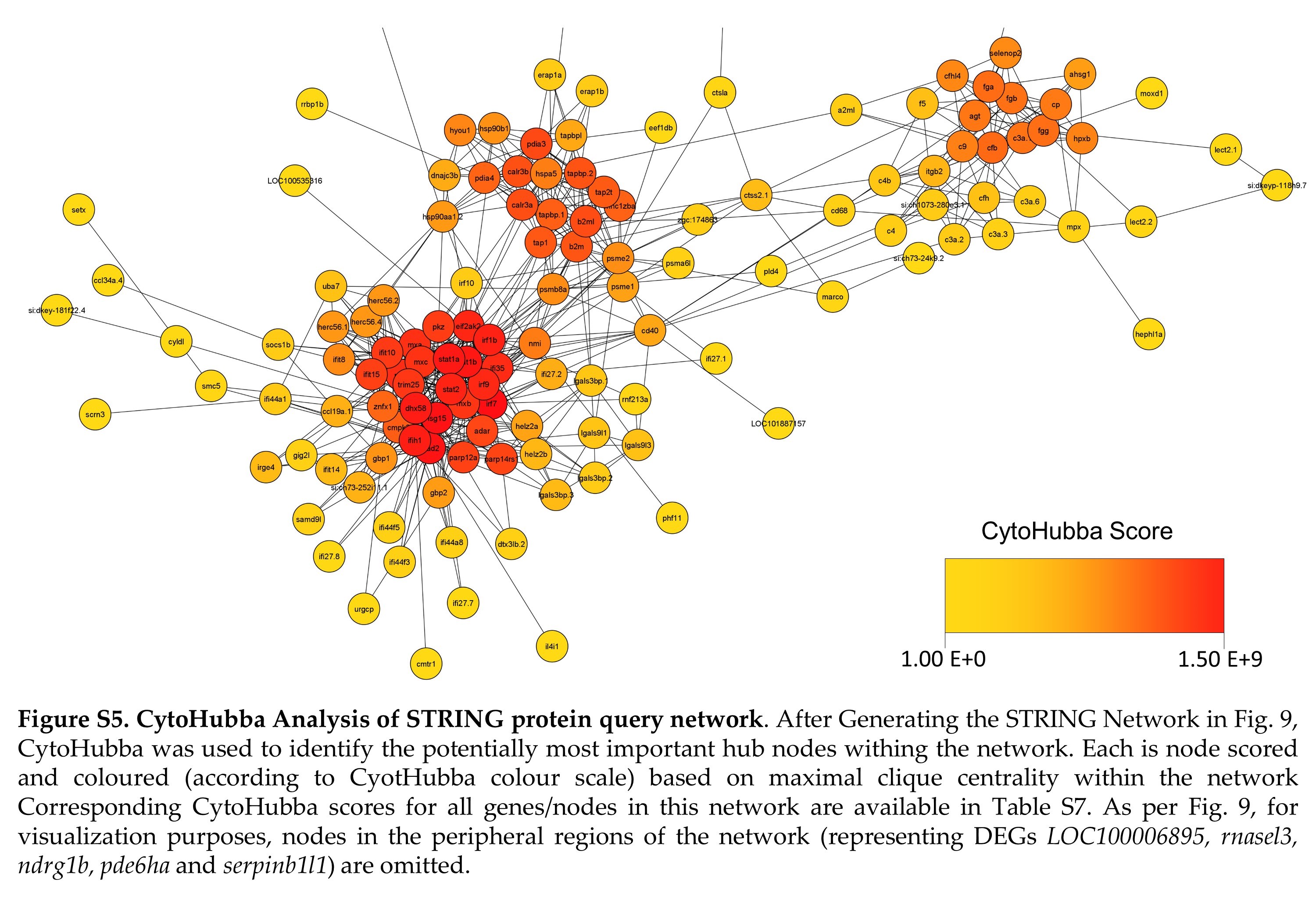

Supplement: Supplementary file 1 [file viruses-15-00768-s001.zip › Figure S5.JPG]

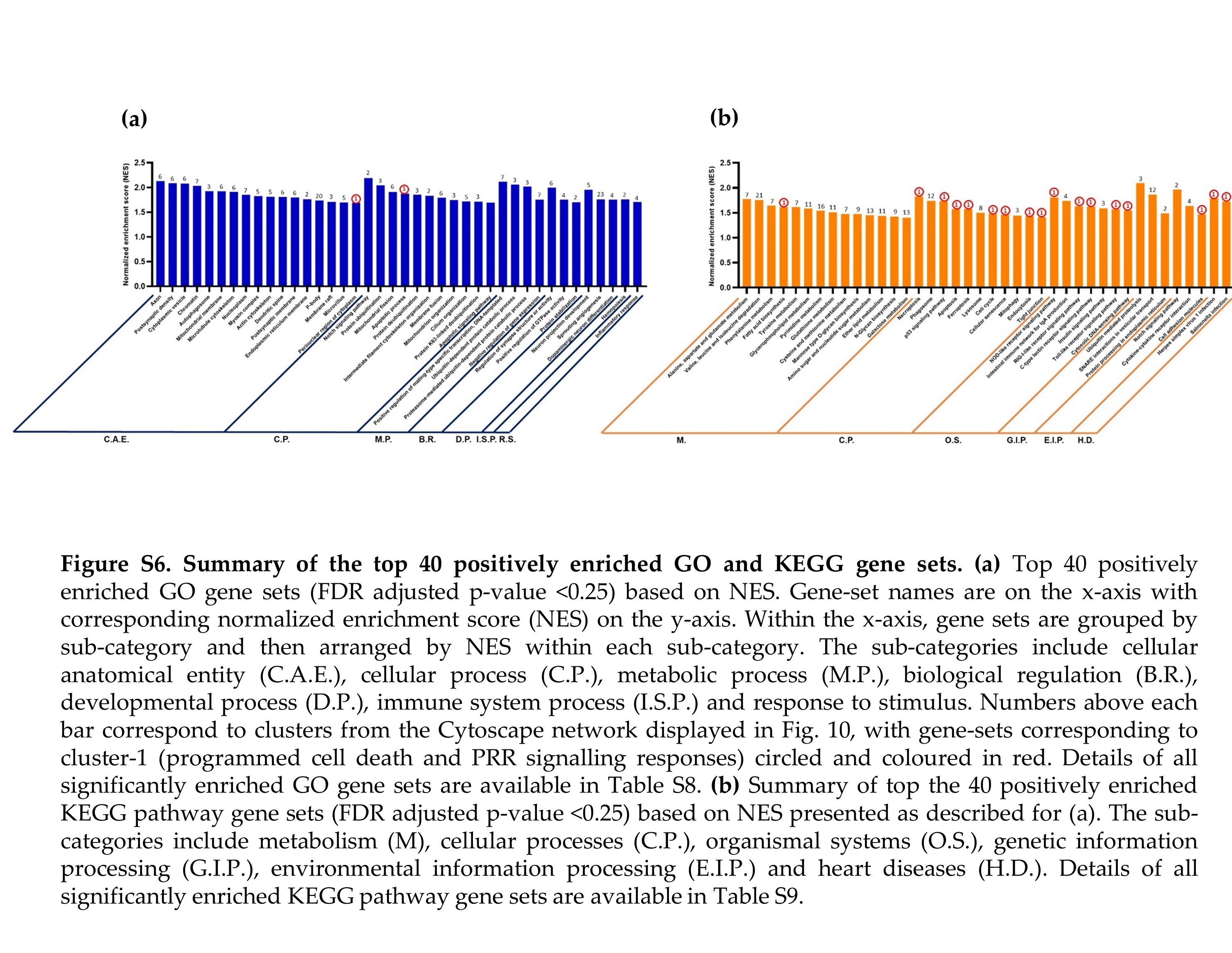

Supplement: Supplementary file 1 [file viruses-15-00768-s001.zip › Figure S6.JPG]

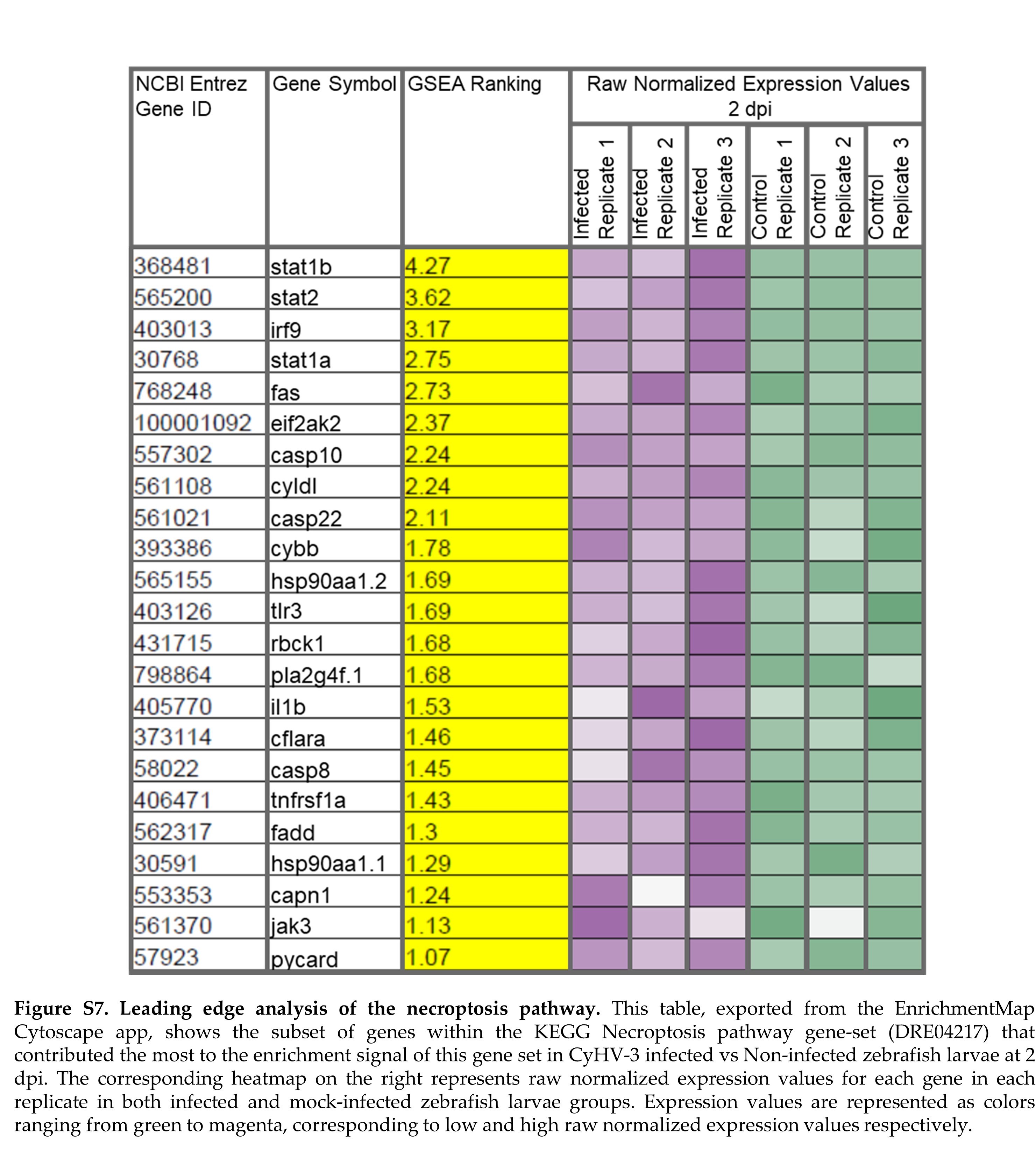

Supplement: Supplementary file 1 [file viruses-15-00768-s001.zip › Figure S7.JPG]

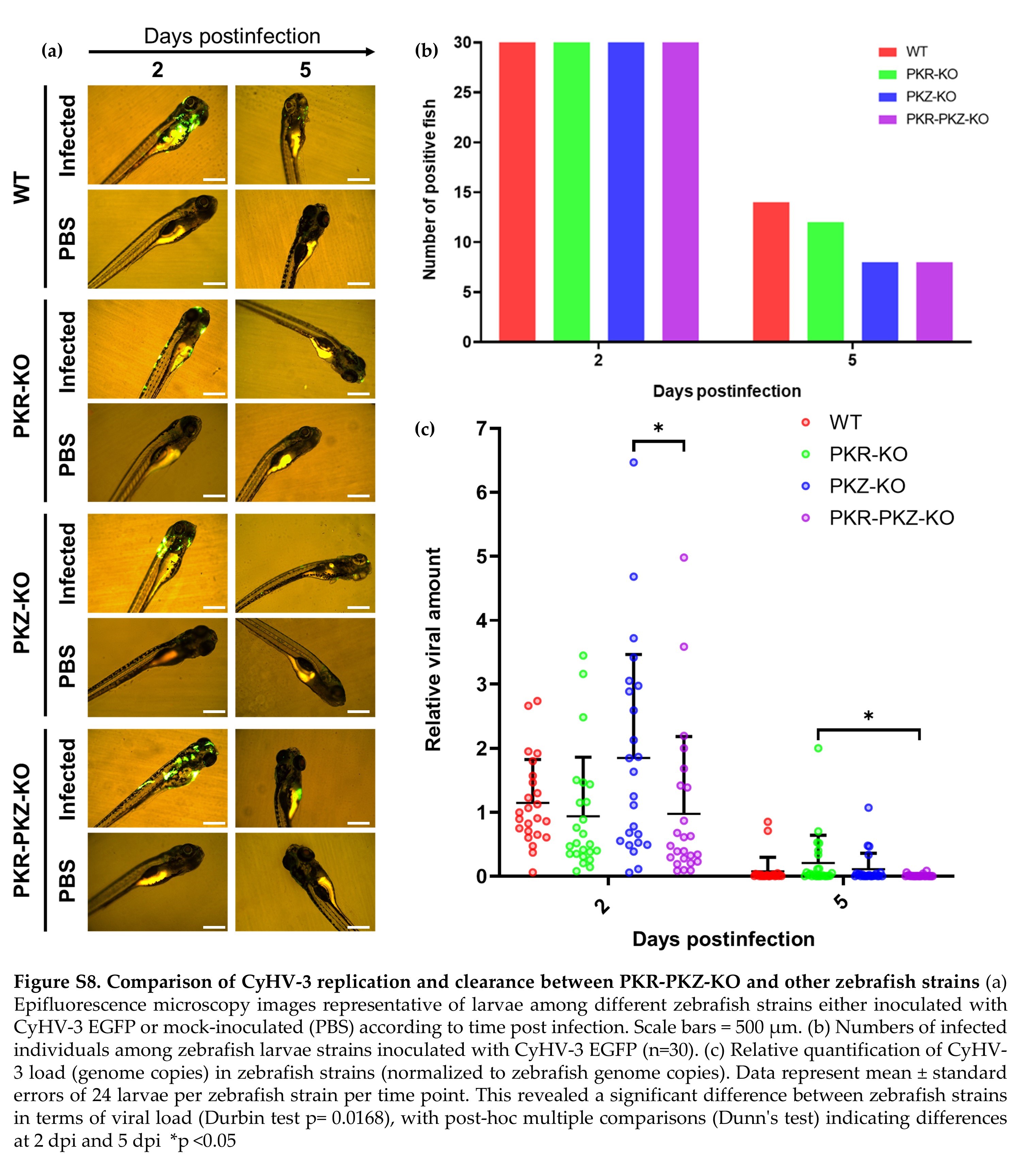

Supplement: Supplementary file 1 [file viruses-15-00768-s001.zip › Figure S8.JPG]
